# Logic and felicity in the face of intellectual disability: linguistic scales and Williams syndrome

**DOI:** 10.1080/15475441.2026.2702526

**Published:** 2026-07-23

**Authors:** Asya Achimova, Julien Musolino, Rennie Pasquinelli, Barbara Landau

**Affiliations:** a Department of Linguistics, University of Tübingen, Tübingen, Germany;; b Psychology Department and the Center for Cognitive Science, Rutgers University, New Brunswick, New Jersey, USA;; c Department of Cognitive Science, Johns Hopkins University, Baltimore, Maryland, USA

## Abstract

Quantifiers (*some, all*), numerals (*two, three*), and logical connectors (*and, or*) belong to the class of logical expressions that convey abstract and complex meaning, denoting properties of sets or relations between sets. In this paper, we show that individuals with Williams syndrome (WS), who display severe deficits in spatial and numerical cognition, and mild-to-moderate intellectual disability, can nevertheless carry out the semantic computations necessary for understanding these expressions. We report data from two experiments testing knowledge of (a) the set-theoretic properties of basic English quantifiers, numerals, and connectors and (b) the logical consequences of these properties (i.e. entailment relations). We compared the performance of 32 adults with WS and 32 neurotypical adult speakers of English. In Experiment 1, people with WS performed significantly above chance (and close to ceiling) on the tests that evaluate the truth-conditional meaning of logical expressions. Moreover, in trials that permit both a strictly logical interpretation and a pragmatically enriched interpretation involving an implicature, speakers with WS and neurotypical adults displayed similar interpretive preferences. In Experiment 2, the WS group scored correctly on more than 90% of trials that evaluated knowledge of the felicity conditions associated with each expression. We interpret these findings as evidence of deep semantic and pragmatic representations in people with WS, notwithstanding their other limitations. We maintain that the relatively small differences in performance between neurotypical adults and individuals with WS are a matter of degree, not of kind. This conclusion allows us to move beyond the question of whether language development in WS is “typical” and instead focus on the underlying computational mechanisms that support language use and understanding in adults with WS. We discuss our results in the context of questions about the independence of language from other cognitive systems, and more generally, the modularity debate.

## Introduction

The study of language in atypical populations offers cognitive scientists a unique tool to understand the human mind. This view fits within an approach that Gleitman and Newport (1995) describe as “changing the learner’s mental endowment” (p. 10). Along with approaches that study outcomes from changing the environment, this approach enables language scientists to search for robust patterns of language acquisition in cases where the internal cognitive representations might change across a variety of learners and learning opportunities. In this paper, we approach the search for robust patterns of language acquisition by asking whether people with Williams syndrome – a relatively rare neuro-cognitive disorder – nevertheless acquire some of the complex patterns of semantic computation known to be mastered by typically developing individuals.

Williams syndrome (WS) is a relatively rare (1 in 7500 live births) neurodevelopmental disorder caused by a micro-deletion of genetic material on chromosome 7q11.23 (Ewart et al., 1993; Strömme et al., 2002), and evidence from their cognitive profile has been long taken as support for the modularity of the mind. Fodor (1983) proposed nine criteria that define modular systems, including domain specificity and characteristic breakdown patterns, also known as “robustness under deficit.” These two criteria are particularly relevant to the study of linguistic abilities in people with WS. Alongside moderate intellectual impairment (mean IQ around 60 and a range between 20 and 106; Ewart et al., 1993) and severe impairment in spatial representations (Bernardino et al., 2013; Landau & Hoffman, 2005; Mandolesi et al., 2009; Mervis et al., 1999), individuals with WS often show fluent and apparently well-structured language (Bellugi et al., 1988, 1994, 2001; Musolino et al., 2010). Importantly, this asymmetry between linguistic and non-linguistic abilities is not found in other atypically developing individuals with comparable levels of intellectual disability, e.g., Down Syndrome (Brock, 2007; Fowler et al., 1994). Together with evidence from individuals with Specific Language Impairment, WS has been argued to offer a case of a double dissociation between linguistic and other cognitive domains (Clahsen & Almazan, 1998; Pinker, 1991, 1997). The double dissociation has been seen as one of the signatures of the modularity of the mind, since it offers the possibility of having one cognitive domain intact (e.g., language), while other modules (e.g., number and space) are significantly impaired, speaking to the criterion of robustness under deficit. One clear criterion for robustness under deficit for the specific domain of language would be evidence of the successful acquisition of complex linguistic representations and computation in the face of an overall intellectual disability.

In this paper, we use the case of logical expressions to examine this claim, asking whether people with WS show knowledge of deep properties of these linguistic elements, consistent with mature typical adult usages. More generally, we seek to understand whether an abstract domain of language – here, logical expressions – can be acquired by individuals with WS, despite their patent deficits in space and number, with the latter most closely associated with the meanings of at least some logical expressions. We further review relevant literature that questions whether the deficits in space and number are as broad as has been originally assumed, and whether the understanding of space and number in people with WS may provide a sufficient basis for mastery of abstract logical expressions. We then interpret the findings in the context of other known skills and deficits, leading to a more nuanced understanding of what the profile of knowledge of logical expressions in WS can tell us about the possible role of modularity in understanding this aspect of language development, whether typical or atypical.

In the following sections, we first survey the empirical literature on linguistic abilities in individuals with WS. We then explicate the linguistic and conceptual complexity of logical expressions, focusing on what people must represent in order to make adult-like judgments regarding the meaning of these expressions, and what we know about patterns of acquisition in typically developing young children. We follow this with two experiments probing knowledge of logical expressions in people with WS as compared with neurotypical adults.

## Background

### Status of linguistic abilities in people with WS

The status of linguistic skills in people with WS has become a topic of considerable debate. While early studies have suggested that people with WS show a high level of verbal ability (Bellugi et al., 1988, 1994), later work has identified deficits in a number of linguistic domains. Thus, in morphosyntax, Karmiloff-Smith et al. (1997) argued that individuals with WS show lack of generalization in the area of agreement morphology. In their studies, even though French-speaking individuals with WS were able to successfully repeat nonce words and were more accurate than controls when they had to produce concordant adjectives both for real and nonce words, they made significantly more errors than the control group. For example, they used a masculine form of the adjective *vert* “green” with a feminine noun *la fourmi* “ant” in a phrase *sous la fourmi vert* (“under the green ant”) instead of the correct form *sous la fourmi verte*. The authors interpreted the asymmetry in performance between these two types of tasks as evidence of morphosyntactic deficits and hence impaired linguistic abilities. Further studies have suggested that morphosyntactic limitations are not restricted to the domain of agreement morphology: upon conducting a meta-analysis of 38 studies and 592 effects, Almería-Morena and Romero-Rivas (2026) concluded that people with WS have worse morphosyntactic skills than typically developing individuals matched by chronological (CA) or mental age (MA).^1^

The lexical skills of people with WS have also been claimed to diverge from typically developing (TD) control groups. In the domain of meaning, Bellugi et al. (1994) questioned the abstractness and depth of semantic representations in WS, noting their tendency to use low-frequency words that may not fit the context appropriately (but see Brock, 2007; Thomas et al., 2006, for rebuttals of this view). The speech of people with WS has been sometimes described as superficially fluent but lacking true understanding, a phenomenon sometimes described as “cocktail-party speech” (Meyerson & Frank, 1987; Udwin & Yule, 1990, 1991). One of the possible causes of this effect might be what Johnson and Carey (1998) referred to as shallow conceptual representations – representations that lack qualitative change which neurotypical children usually show in their conceptual development. Conceptual change involves a deep revision of concept structure which can result either in the abandonment of a simpler concept or an emergence of a more general concept that projects its features to its branches. For example, young children differentiate between animals and people, and only later form a new category of living beings that encompasses animals, people, and plants. Johnson and Carey (1998) demonstrated that adolescents and adults with WS possessed general knowledge about the relevant concepts, such as living beings, that corresponded to the level of a 10-year-old TD child (e.g., they were able to produce names for different types of animals and objects and surpassed younger neurotypical children in the range of facts about animals and people they knew). However, their conceptual understanding of biological concepts, such as life, death, and people-as-one-animal-among-many remained at the level of TD preschoolers. For example, WS individuals lacked a category of living beings that included both animals and plants. These properties of their conceptual system likely surface as reduced lexical-semantic skills in tasks probing the associations between different concepts.

Possibly related to the conceptual deficits, people with WS show poor performance on non-literal language tasks that assess the structure of their mental lexicon (Mervis & John, 2008, 2010; Sepúlveda & Resa, 2024). A recent meta-analysis of 42 studies suggests that overall, speakers with WS have worse lexical-semantic skills than neurotypical children and adults matched either by chronological or mental age (with the latter groups being considerably younger than the groups with WS; Romero-Rivas et al., 2023). More specifically, the meta-analysis revealed that while people with WS were not different from neurotypical control groups on vocabulary tasks (e.g., picture naming), they showed worse semantic integration (e.g., ERP-response to semantically anomalous words in context, judging whether words or word/image pairs forming a category belong together), semantic memory organization (e.g., spontaneously naming words that belong to a category, false memories during recall of semantically related words), and verbal working memory tasks. The interpretation of these findings, however, is clouded by the heterogeneity of the tasks evaluated and the fact that different studies rely on different control groups matched sometimes by chronological age and sometimes by mental age. One aspect, however, remains clear: with very few exceptions,^2^ these previous studies have largely focused on open class words (e.g., nouns, verbs, and sometimes adjectives) specifically evaluating the connectedness and structure of the mental lexicon in people with WS, which possibly relates to their overall conceptual structure rather than difficulties with meaning computation.

The underlying computational system that supports language production and comprehension has been the focus of a relatively few studies. Thus, Clahsen and Almazan (1998), as well as Pinker and Ullman (2002), originally argued for a spared computational system in people with WS in the domain of past-tense formation, supported by spared production of tense for regular verbs (guided by a rule-based system), but not irregulars (involving associative memory, which is impaired in WS). The overall findings of these studies, however, were contested. Later work failed to replicate the original reported asymmetry between regular vs. irregular past tense formation with a larger sample (*n* = 21; Thomas et al., 2001), and the original findings of the asymmetry have been criticized because of the very small number of participants (*n* = 4). More recent work has found no sharp asymmetry in the acquisition of regular and irregular past tense forms in adolescents and adults with WS (Pasquinelli, 2024), suggesting a single underlying mechanism that is like that of typically developed individuals (see Albright & Hayes, 2003). Pasquinelli (2024) further demonstrated that adolescents and adults with WS show similar patterns of generalization to those of neurotypical children who were matched to the WS participants by mental age. In experiments that involved producing past tense forms for high and low-frequency verbs, people with WS showed sensitivity not only to the differences between regular and irregular past tenses, but they also recognized patterns within the class of irregular verbs, just like neurotypical children did, producing similar overregularization errors (Pasquinelli, 2024). Importantly, for the present work, errors on those tasks have been related to associative memory capacity and thus may reflect deficits in cognitive systems that lie outside language.

In the early studies on tense, similarities in performance between individuals with WS and TD children matched by mental age have been interpreted as an indication of an “intact” underlying linguistic system in WS (Clahsen & Almazan, 1998; Pinker, 1991; Rossen et al., 1995). However, as Karmiloff-Smith (1997, 1998) argued, even if people with WS do reach the same level of linguistic skills as TD children matched by mental age, this performance level does not necessarily signal “intact” skills, since adults with WS may have developed these skills through other compensatory processes, e.g., relying on good auditory memory instead of acquiring deep abstract linguistic patterns. This interpretation is consistent with a “neuroconstructivist” view which maintains that atypical brain and cognitive development necessarily follow different paths from typical development, so one cannot simply measure the end-state, but must also measure the rate of development and the age at which the end-state is achieved. Strong phonological memory has been invoked to explain performance on some semantic measures, such as the Category Test for Semantic Fluency (Semel et al., 1987), where individuals with WS surpassed MA matches (Volterra et al., 1996). These findings informed the “semantic-phonological imbalance” hypothesis (Thomas & Karmiloff-Smith, 2003) which attributed mixed performance on semantic tests to shallower conceptual representations of people with WS (Johnson & Carey, 1998; Romero-Rivas et al., 2025). Karmiloff-Smith further questioned whether ceiling or close to ceiling performance of adolescents and adults with WS on language tasks can in principle be seen as evidence of complex linguistic knowledge, since that knowledge is readily mastered by TD children by the age of 7 years.

In this paper, we challenge this argument, turning to the example of logical expressions, which, though they appear simple, actually involve abstract knowledge and complex computation. We further emphasize that just because some aspect of linguistic knowledge matures early does not mean this should be attributed to the simplicity of the principles that underlie some aspects of language use. As has been demonstrated for many linguistic phenomena, understanding even seemingly “simple” sentences often relies on complex semantic computation. Taking the interaction of logical disjunction and negation as an example, Musolino and Landau (2010, 2012) pointed out that the ability to correctly understand sentences, such as “The cat who meowed will not be given a fish or milk” vs. “The cat who doesn’t meow will be given a fish or milk” requires knowledge of intricate syntactic and logical principles. Typically developing 6-year-olds show near-ceiling performance on the tasks that Musolino and Landau used to test understanding of such sentences (and so do adults with WS). Rather than casting doubt on the complexity of the underlying computation, the ultimate mastery of such structures indicates that both TD children and individuals with WS must have the capacity to represent the linguistic structures needed to interpret such sentences. It is exactly the presence of such linguistic knowledge that we seek to test in individuals with WS. Examining the final achievements of acquisition can reveal whether people with WS have achieved a similarly complex computational system to individuals who are developing typically. We return later to the question of whether there are other, simpler mechanisms which might account for our data on linguistic knowledge among individuals with WS, and we will argue that no viable alternative mechanism has been proposed to date.

In this work, we turn to the words that form a closed class – a class of lexical expressions that capture a fixed and restricted range of meanings. Examples of this class include prepositions (e.g., *in, on*), connectives (e.g., *and, or*), numerals (e.g., *two, three*), and quantifiers (e.g., *some, all, not all, none*). Among these, logical expressions, such as quantifiers and connectors, carry abstract meaning, encoding fundamental relations between objects, agents, and properties. In the next section, we turn to the meaning of logical terms and illustrate its underlying complexity. We then argue that investigating how people with WS understand these terms may offer a new way to tap into the depth of their semantic representations by testing the computational system that underlies knowledge of language. If individuals with WS show mastery of deep semantic principles, this would lend support to the idea that properties of at least some linguistic terms that engage deep aspects of semantic computation can be acquired despite overall intellectual disability, showing robustness under deficit-one of the hallmarks of modules (Fodor, 1983). Turning to the study of logical operators thus offers a window into meaning computation that is more independent of conceptual development than other aspects of vocabulary might be.

### The meaning of logical terms

Logical terms (quantifiers, connectors, numerals, and other types) belong to closed class lexical expressions which encode a fixed set of logical meanings. Whereas open class words evolve over time, with some disappearing and others entering the language, closed class words exhibit a comparatively stable inventory. One of the most well-studied classes of logical expressions in semantics is the class of natural language quantifiers, words such as *some, all, many, few*, and others.

Natural language quantification takes a special place in the study of meaning: it reveals the complexity of computations that underlie the interpretation of utterances. The universal nature of quantification likely reflects its core role in communication. At the same time, interpreting even the most mundane statements that involve a quantifier, such as *some*, requires knowledge of an intricate array of interacting lexical, syntactic, semantic, and pragmatic principles. The search for an empirically adequate way to capture the meaning of these terms has shaped entire fields in the study of meaning, most notably formal semantics and pragmatics.

The meaning of quantifiers is more abstract than the meaning of referential noun phrases: while referential noun phrases, such as *Ann* and *Sarah* in (1a), refer to individuals, quantificational phrases, such as *some students* in (1b) denote relations between sets (Barwise & Cooper, 1981; Montague, 1973).^3^ Thus, the quantifier *some* in (1b) means that the intersection of the sets of students and individuals that received an A is non-empty, while the quantifier *all* in (1c) applies when the set of students is a subset of all individuals that received an A.

(1) a. Ann and Sarah received an A.
b. Some of the students received an A.
c. All of the students received an A.

The view that quantifier meaning is defined in terms of set relations that they capture grew out of the predicate logic tradition, where the meaning of *some* is described using the existential operator. For example, the truth conditions of a sentence such as (1b) can be captured using the expression in (2) that defines the logical meaning of the quantifier: the sentence in (1b) is true in case there is at least one student who received an A.

(2) ∃x Student (*x*) & Received an A (*x*).
  logical formula

The logical meaning of quantifiers, as defined in formal logic and shown in (2), has been termed “semantic,” since it represents truth conditions under which a sentence with this quantifier is true, and in Gricean pragmatics refers to “what is said” (1975). The semantic/pragmatic distinction has been widely adopted in the acquisition literature (e.g., Katsos, 2008; Ozturk & Papafragou, 2015).

The logical (semantic) meaning of the example (1b) is shown in (3a), where *some* is interpreted as “some and possibly all.” However, when interpreting such statements, speakers often pragmatically enrich the semantic meaning of the quantifier by computing an implicature, as shown in (3b). This pragmatic enrichment is possible due to scalar properties of quantifiers. Quantifiers, such as *some*, belong to a class of words that can form scales ordering elements according to their strength (Horn, 1972). In a situation where two statements that involve different scalar terms from the same scale are true, the statement containing the entailing quantifier will always be more felicitous, or “pragmatically appropriate.”^4^ Crucially, strength here is defined in terms of entailment relations such that given two scalar terms, say *some* and *all*, the entailing term, in this case *all*, is the stronger term. Cooperative speakers, according to Grice (1975), are expected to follow the maxim of quantity by providing as much relevant information as required, but no more. For scalar terms, it means choosing the strongest possible term on the scale. If a speaker does not use a stronger term, it means that the stronger term does not apply, and the listener computes a relevant scalar implicature (Horn, 1972), pragmatically enriching the meaning of a weaker term.

(3) a. Some and possibly all students received an A.
  logical reading
b. Some but not all students received an A.
  pragmatically enriched reading

We can observe similar scalar properties for other types of quantifiers. For example, for the pair <*not all, none*>, *none* is the stronger term. If a speaker produces a sentence in (4), she likely implies that the term *none* does not hold. However, at least in principle, the weaker term *not all* can have a logical reading, as in (4a) and a pragmatically enriched reading, as in (4b).

(4) Not all of the apples are red.
a. Not all and possibly none of the apples are red.
  logical reading
b. Not all but not none of the apples are red.
  pragmatically enriched reading

The understanding of entailment relations is complicated by the fact that scalar terms differ in their tendency to give rise to scalar implicatures and consequently may have a different likelihood to be interpreted pragmatically (Baker et al., 2009; Beltrama & Xiang, 2013; Orr et al., 2024; Sun et al., 2018; Van Tiel et al., 2016). Thus, experimental evidence suggests that the quantifier *some* is often interpreted pragmatically to mean “some but not all” while the logical operator *or*, relative to *some*, is more likely to have a logical reading as “either *x* or *y* and possibly both,” as in (5a), than an implicated reading, as in (5b) (Geurts, 2010; Van Tiel et al., 2016).

(5) A cupcake or a star is painted on the wall.
a. A cupcake or a star, and possibly both are painted on the wall.
  logical reading
b. A cupcake or a star, but not both are painted on the wall.
  pragmatically enriched reading

Numerals, such as *two* and *three*, in turn, strongly depend on the context in their interpretation. Numerals are taken to have lower-bound semantics: for example, two means “at least two,” as in (6a).

(6) John has two children.
a. John has two and possibly more children.
  logical reading
b. John has two but not more than two children.
  pragmatically enriched reading

The upper-bound meaning “two but not more than two,” as in (6b) likely stems from an implicature. An important property of implicatures is that they can be canceled. This indeed appears to be possible for examples with numerals, as Horn (1989, p. 215) illustrates, shown here in (7):

(7) Pat has three children and possibly four.
  three children and for all I know four.
  three children if not four.
  three or even four children.
  three, indeed four children.

Whether the exact interpretation is the result of pragmatic inference or this meaning is encoded lexically remains a topic of theoretical debate (see Spector (2013) for an overview). Under cognitive load, speakers often interpret numerals exactly (Marty et al., 2013; for acquisition data see also Musolino (2004)), suggesting that this meaning may be encoded lexically. However, we will abstract away from this debate and use the terms “logical reading” and “pragmatically enriched reading,” as in (6a-b) to preserve parallelisms to other examples.

The likelihood of deriving a pragmatic meaning further depends on contextual factors (Degen, 2015) as well as on structural properties of the sentences in which they occur (Schuster et al., 2020). Reasoning about the meaning of quantificational terms is additionally shaped by one’s expectations about the knowledgeability of the speaker, with the semantic meaning of a quantifier being more easily accepted if the speaker is not fully knowledgeable (Breheny et al., 2013; Goodman & Stuhlmüller, 2013; Zhang et al., 2023).

So far, we have considered a range of factors that either favor or impede the calculation of scalar implicatures, and, therefore, access to the pragmatically enriched interpretation of logical expressions. In this overview, we have adopted an implicit assumption that the pragmatic meaning is defined as an *enrichment* of the semantic meaning since it involves an additional inference (scalar implicature) that is derived on top of the logical meaning. However, it remains debated whether this theoretical assumption has empirical support. One way to approach the question of which of the two meanings is primary is through the study of the acquisition of logical expressions. In this literature, the relative difficulty of making pragmatic inferences for logical expressions with scalar properties is taken as evidence that children access the semantic meaning first and the computation of pragmatic inferences requires additional resources. For example, children have been shown to accept the use of *some* as “some and possibly all,” thus revealing the primacy of semantic, or logical meaning. Noveck (2001) brings together evidence from the use of *some* in generic contexts (Smith, 1980) and the use of logical operator *or* in contexts where *and* also holds (Braine & Rumain, 1981; Paris, 1973). This work has shown that 70% of the 10-year-old children (compared to 33% of adults) found the logical meaning of the term compatible with contexts where the stronger term holds, such as “Some giraffes have long necks” (such contexts are sometimes termed “underinformative”). A more recent systematic review further confirms that scalar implicature derivation is cognitively effortful in adults, albeit the effects are rather weak (Nys et al., 2024), supporting the “primacy of logical meaning” view. At the same time, the primacy of logical meaning is difficult to reconcile with the fact that at least for some quantifiers, their logical use is rarely present in the input. Large-scale experiments in language production suggest that speakers normally do not produce terms such as *some* to refer to situations where *all* is true (van Tiel et al., 2021). These patterns give rise to an acquisition puzzle: how do speakers acquire the semantic meaning of these terms if they rarely, if ever, receive direct evidence for it in their linguistic input? We will return to this acquisition puzzle in the General Discussion.

To summarize, we have focused on logical expressions, such as quantifiers, numerals, and logical connectors, as a type of expressions that convey abstract meaning. Given the complexity of their meaning, both logical (semantic) and pragmatically enriched via an implicature, we now ask whether individuals with WS are able to interpret sentences with such expressions. If semantic representations in people with WS are shallow, we would expect them to struggle with the meaning of logical expressions, since it requires a set of abstract semantic principles to be operable. However, if the understanding of set relations and logical entailment is intact (i.e., similar to neurotypical adults), we would expect that the representation of core aspects of logical expressions – both their logical and perhaps even pragmatically enriched meanings – should be preserved in adolescents and adults with WS. In Experiment 1, we ask whether individuals understand the two aspects of meaning of logical expressions related to their truth conditions and entailment patterns. In Experiment 2, we ask whether the same individuals with WS further recognize felicity conditions associated with the use of these expressions.

## Experiment 1: core meanings of logical expressions

### Participants

We tested 32 participants with Williams syndrome (23 F; *M* age = 21;0 years, range = 11;10–47;6 years) and 32 neurotypical adults as the control group (14 F, range = 18–60 years). Within the WS group, there was a cohort in which participants were tested individually in our lab (*N* = 12, *M age* = 16;4 years; range = 11;10–21;11) as well as a cohort in which participants were tested in the same experiments via videoconferencing (*N* = 20, *M age* = 23;4 years; range = 12;8–47;6). Similarly, within the neurotypical adult group, there was one cohort of undergraduate participants who were tested individually in our lab (*N* = 12)^5^ and a second in which participants were tested via videoconferencing on the Prolific platform (*N* = 20, *M* age = 38;4 years, range = 25;0–60;11).

Individuals with WS were recruited through the Williams Syndrome Association, social media groups, and personal contact from the list of participants who took part in previous experiments carried out in our lab. All of these individuals, except for one, received a positive diagnosis using a FISH (fluorescence in situ hybridization test; Ewart et al., 1993). Participants were additionally tested on verbal and non-verbal reasoning parts of the Kaufman Brief Intelligence Test, which is a standardized intelligence test that has relatively few spatial items and hence does not unfairly penalize people with WS for their severe spatial deficit. More specifically, the in-lab cohort completed the first edition of the KBIT (KBIT, Kaufman & Kaufman, 1990) which included two sub-scales: verbal knowledge (*M* = 41.4, *SD* = 6.24) and non-verbal reasoning (*M* = 21.7, *SD* = 4.03). The online cohort, who participated in the experiment at a later time, completed the second edition of the test (KBIT-2, Kaufman & Kaufman, 2004) which included two verbal sub-scales (verbal knowledge and riddles, composite raw score *M* = 57.0, *SD* = 18.8), as well as a non-verbal sub-scale (*M* = 23.0, *SD* = 6.84). Raw scores on the verbal subtests were in the range of other studies of individuals with WS (Lakusta & Landau, 2005; O’Hearn et al., 2005; Pasquinelli, 2024). There were no significant differences between the in-lab and online cohorts of WS or control participants in terms of their performance on different types of trials that we detail below; we report these comparisons, as well as summaries of demographic information (Table A.1) in the Appendix A.

The experimental protocol was approved by the Institutional Review Board of Johns Hopkins University and the Ethics Committee for Psychological Research at the University of Tübingen. We obtained written consent from all the participants or their caregivers, whenever obtaining a written consent from a participant was not possible. Participants received compensation for their participation at the rate of $12 per hour. People with WS attended two sessions: the main session where they completed the experiments, which lasted 1 hour with a short break between experiments, and an additional session that lasted 30 min and included KBIT (intelligence) testing. Neurotypical adults were tested in only one main experimental session which lasted 1 hour; no intelligence tests were carried out with this group since we expected the control group of neurotypical adults to perform at ceiling, so a correlation with intelligence scores would not have been meaningful.

### Design, stimuli, and procedure

We tested participants’ interpretation of sentences containing logical expressions using the Truth Value Judgment Task (Crain & Thornton, 1998) – a technique proven to be successful in assessing the interpretation of a broad range of linguistic constructions in typically developing children (Lidz & Musolino, 2002; Musolino & Lidz, 2003, 2006; Musolino et al., 2000), as well as individuals with WS (Musolino et al., 2010; Zukowski, 2001). All participants were tested on five terms, each corresponding to a specific logical expression: *all, not all, some, or*, and *two*. The terms *not all, some, or, two* were chosen because they each represent the weaker term on a given scale: <*not all*, *none*>, <*some, all*>, <*or, and*>, and <*two, three* >. The quantifier *all* acted as a control since we tested for knowledge of the meaning of *not all*.

**Figure 1.**
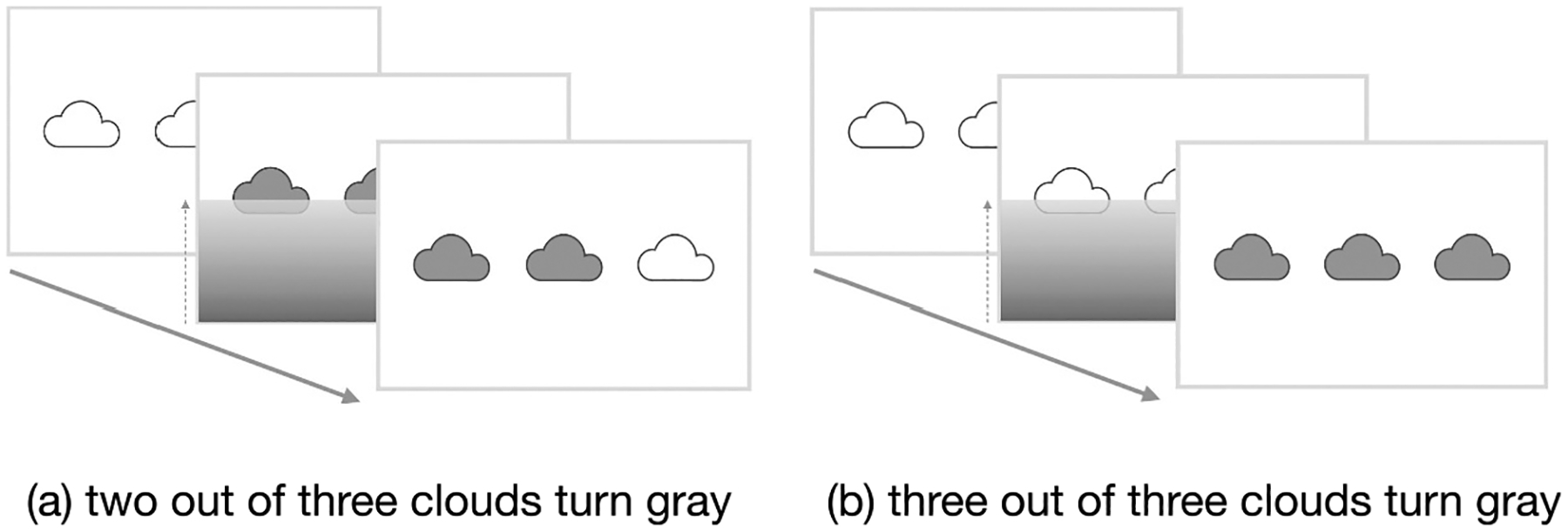
Experiment 1, sample trials. Participants watch an animation accompanied by the statement: “I bet that all of the clouds will turn gray. Let’s see! I said that all of the clouds will turn gray. Am I right?” If participants know what the quantifier *all* means, they are expected to say “no” in the scenario “a” and “yes” in the scenario “b”.

The trials were structured in terms of predictions and outcomes (see Figure 1). For the trials with the terms *some, all, not all*, and *two*, participants first saw three objects displayed on a computer monitor (e.g., circles, squares, stars). Prerecorded statements contained predictions about a change that the objects were about to undergo, and either some, none, or all of the objects then underwent the change.^6^ Trials with *or* were slightly different: participants first saw an empty screen and heard a prediction about the objects that should appear on the screen. They then observed that either zero, one, or two of the mentioned objects appeared on the screen. This structure of the trials made statements with *or* more natural. For all terms, participants were asked to judge, based on the final image, whether the speaker’s prediction was correct or not.

An example of two test trials for the quantifier *all* is shown in Figure 1. In this scenario, participants first saw a screen featuring three white clouds and heard a statement “I bet that all of the clouds will turn gray.” Then, a gray screen covered the white clouds and then uncovered an image where in “a” two out of three clouds turned gray and in “b” three out of three clouds turned gray. The recorded voice then asked “Am I right?” Correct responses to the question were “no” in the scenario “a” and “yes” in the scenario “b.”

**Figure 2.**
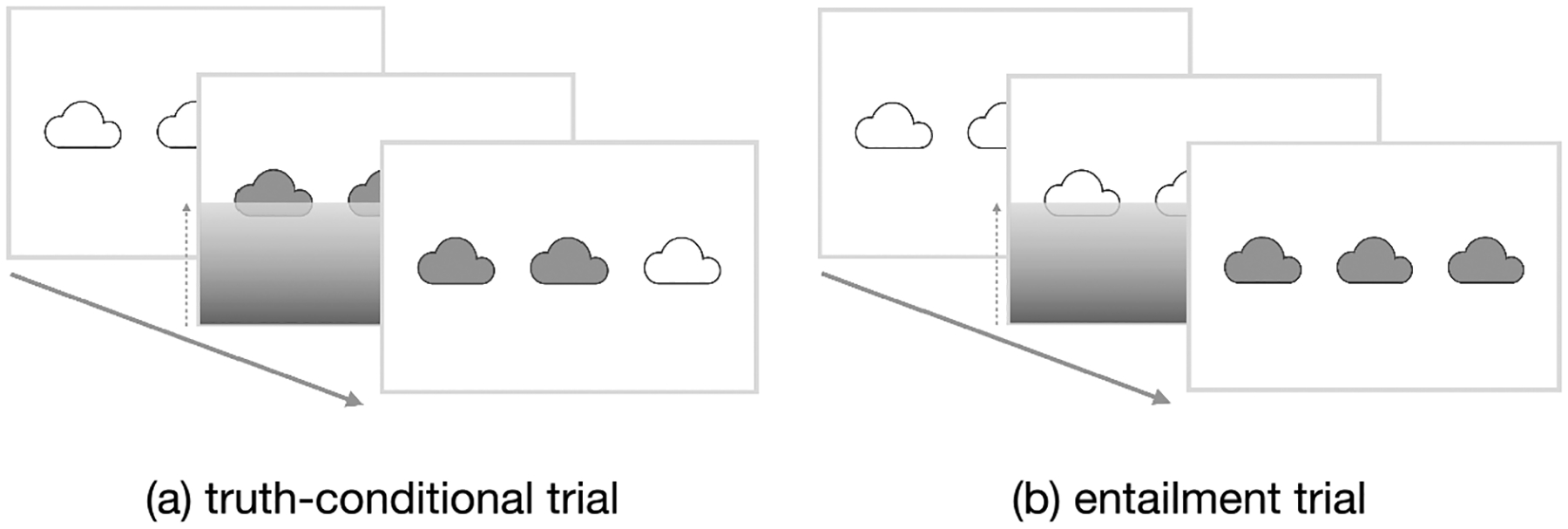
Truth-conditional and entailment trials for the quantifier *some*. Participants evaluated the prediction “I bet that some of the clouds will turn gray.” Their task was to answer the prerecorded question “Am I right?” For the truth-conditional trial in panel (a), responding “yes” is the correct answer. For entailment trials (b), responding “yes” indicates that the participant interpreted the quantifier logically as “some and possibly all,” and responding “no” means that they interpreted the term pragmatically as “some but not all”.

The experiment contained two types of trials. *Truth-conditional* (tr) trials targeted the core meaning of logical expressions – the conditions under which the sentences with these expressions become true and false.^7^ These trials featured scenarios where the speaker’s prediction was true or false. Here, we evaluated whether participants were able to identify true and false predictions. *Entailment* (ent) trials, in turn, featured statements that were ambiguous between a logical reading and a pragmatically enriched reading. Under one of the readings the prediction was true, while under a different reading it was false. Saying that a prediction was true signaled a logical interpretation, while saying that the prediction was false revealed a pragmatic interpretation of the target logical expression. Figure 2 shows an example of a truth-conditional (a) and an entailment trial (b) for the quantifier *some*. For both the truth-conditional and entailment trials, participants evaluated the statement in (8).

(8) Some of the clouds will turn gray.

In the truth-conditional trial (panel “a”), since two of the objects turned gray, the participant should answer “yes” to the prerecorded question “Am I right?” In the entailment trial (panel “b”), three out of three clouds turned gray, and a “yes” answer means that *some* is interpreted logically (“some and possibly all”), and “no” indicates that it is interpreted pragmatically (“some but not all”).

Table 1 summarizes for each combination of a logical expression and an outcome (i.e., how many objects underwent the announced change) which type of trial (tr or ent) that combination constitutes.

Table 2 focuses on entailment trials and shows what interpretations correspond to the logical and pragmatically enriched readings for the relevant terms.^8^

The five logical expressions were each paired with three possible outcomes, with four items per combination of expression and outcome, resulting in a total of 60 test trials per participant (5 logical expressions × 3 outcomes × 4 items). For each expression except *all*, two outcomes made the trials truth-conditional, and one outcome categorized it as an entailment trial. For *some, not all, or*, and *two*, participants completed 8 truth-conditional and 4 entailment trials. Since the quantifier *all* had no entailment trials, participants completed 12 truth-conditional trials. We blocked trials by logical expression in order to reduce possible switching costs, since people with WS often show reduced visual (O’Hearn et al., 2009) and verbal working memory capacity (Romero-Rivas et al., 2023). All trials within each block as well as the order of blocks were randomized.

### Results

We discuss truth-conditional and entailment trials separately in the analysis below. For truth-conditional trials, we first evaluated whether the probability of choosing the correct answer depends on the type of logical expression and participant group. We further calculated the number of correct responses (replying “yes” when the statement is correct and “no” when the statement is wrong). We then evaluated whether for each of the logical expressions, participants performed above chance (i.e., greater than 50% correct). For entailment trials, we evaluated whether the probability of accessing a logical interpretation depends on the type of logical expression and group, as well as whether this probability is different from chance (again, 50% was chance performance suggesting no clear preference for either a semantic or a pragmatic interpretation). Thus, for both kinds of trials, we asked whether the performance of WS individuals was similar or different from the control participants and whether it was above chance.

### Truth-conditional trials

In a binomial mixed-effects model, we tested whether group and type of logical expression predicted the response (correct vs. incorrect); we further included random intercepts for participants and items. For all models reported in this paper, we used a maximal random effects structure that still converged (Barr et al., 2013). The models were evaluated using the package “lmerTest” (Kuznetsova et al., 2017) implemented in R (R Core Team, 2023). We applied sum-coding for the type of logical expression which allowed us to compare the performance on each of the logical expressions to the grand mean; for the group variable, we used treatment coding with the control group set as the reference level. The analysis showed that people with WS were less accurate than neurotypical adults (*b* = −2.20, *SE* = 0.30, *z* = −7.47, *p* < .01; full model output can be found in the Appendix, Table B.1). Due to ceiling effects and low variance in the control group, we were unable to fit a model with the interaction term. Therefore, we estimated the effect of group for each logical term separately using a set of binomial mixed-effects regressions; all the models included random intercepts for participants and items; treatment coding was used for the factor group with the reference level set to the control group. To control for multiple comparisons (5 tests), we applied the Holm correction to the *p*-values. Results showed no significant group differences for *all* (*b* = −1.78, *SE* = 1.56, *z* = −1.14, *p* = .762); *or* (*b* = −2.50, *SE* = 1.18, *z* = −2.12, *p* = .034); *some* (*b* = −1.67, *SE* = 1.72, *z* = −0.97, *p* = .762); and *two* (*b* = −0.72, *SE* = 1.86, *z* = −0.39, *p* = .762). For the negative quantifier *not all*, people with WS were less accurate than the control group (*b* = −3.44, *SE* = 0.93, *z* = −3.70, *p* < .01).

**Figure 3.**
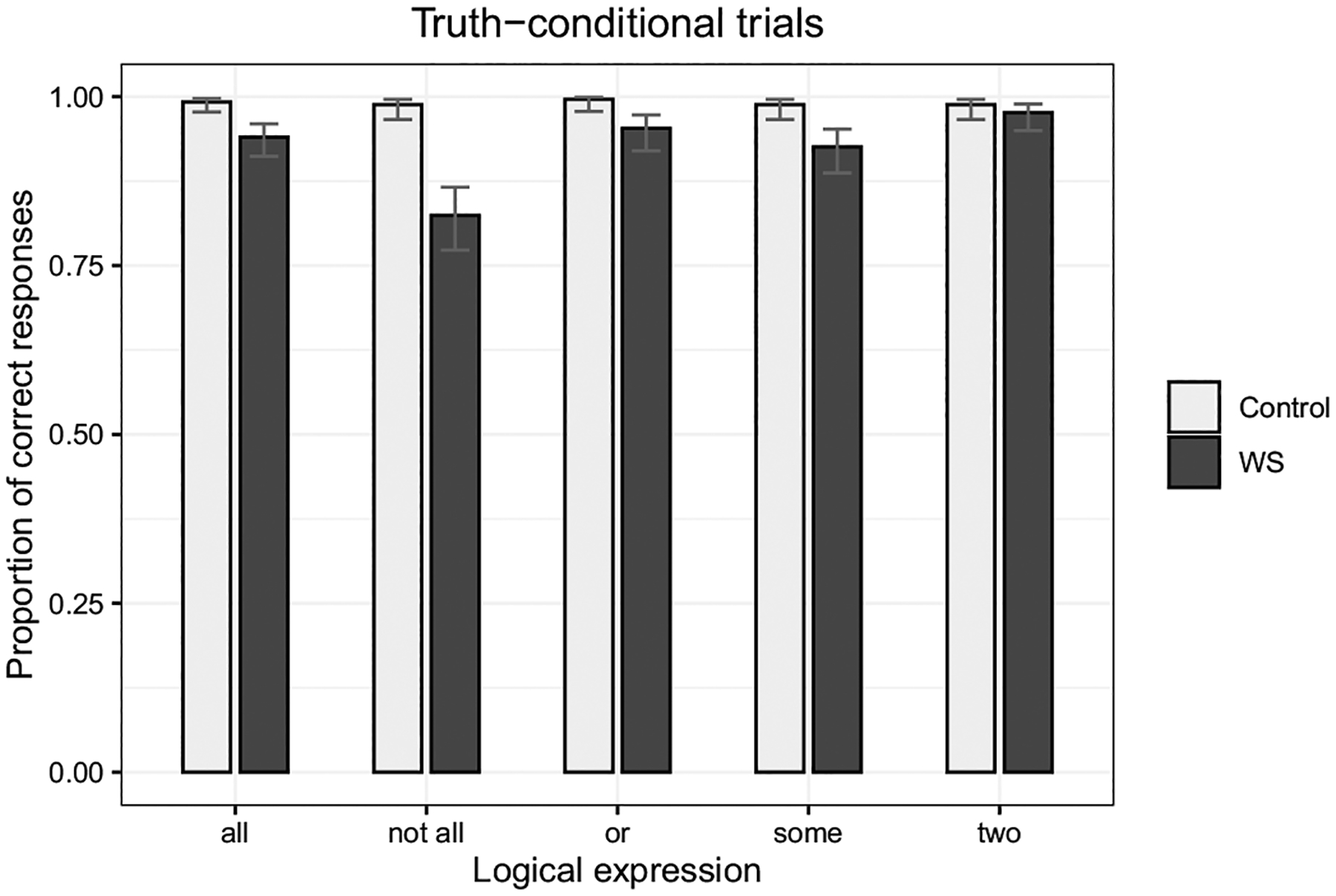
Proportion of correct responses by WS and control participants for the five logical expressions in truth-conditional trials. Correct responses for each outcome and expression are defined in Table 1. Error bars represent 95% binomial confidence intervals.

To investigate whether people with WS show evidence of the principles of semantic computation that underlie their comprehension, we now compare their performance to chance levels. Across all terms taken together, all speakers with WS performed significantly above chance (the null hypothesis that performance is not different from a chance level of 50% can be rejected at the *p* < .05 level with a binomial exact test if a participant correctly responded to 28 trials out of 44). As expected, neurotypical adults performed at ceiling (98.8 −99.2% correct; Figure 3).

In more detail, people with WS scored above 90% correct (93 − 97%) on all logical expressions except for *not all* where they reached more than 82% correct. We applied a binomial exact test to calculate whether each participant in the WS group performed significantly above chance for each of the terms. For *not all*, 66% of participants in the WS group performed significantly above chance (the null hypothesis can be rejected at *p* < 0.05 level if a participant gave at least 7 correct answers out of 8). More specifically, of the 32 participants, 16 correctly responded to all of the questions that contained *not all*, and only six participants correctly completed fewer than 50% of the trials.

### Effect of demographic variables on the probability of correct responses in truth-conditional trials

For the WS group, we further analyzed whether demographic characteristics predict performance in truth-conditional trials. We ran a binomial mixed-effects regression where the type of response (correct vs. incorrect) served as the dependent variable, and age in years, KBIT raw verbal and non-verbal scores were treated as continuous predictors.^9^ Since the in-lab and online cohorts completed different versions of the KBIT, we report regression results for these groups separately. For the in-lab cohort, the analysis revealed a small negative effect of age on the response (*b* = −0.11, *SE* = 0.04, *z* = −3.05, *p* < .01), and a small but significant positive effect of the verbal KBIT score (*b* = 0.10, *SE* = 0.02, *z* = 4.42, *p* < .001). The non-verbal KBIT score did not significantly predict the response (*b* = 0.08, *SE* = 0.05, *z* = 1.69, *p* = .91; please see Appendix, Table B.2 for the full output table and model syntax). For the online group, we found a similar pattern: there was a small negative effect of AGE (*b* = −0.11, *SE* = 0.03, *z* = −3.06, *p* < .01), and a positive effect of verbal score (*b* = 0.11, *SE* = 0.02, *z* = 5.29, *p* < .001), as well as no effect of the non-verbal score (*b* = 0.01, *SE* = 0.04, *z* = 0.31, *p* = .760). Table B.3 in the Appendix shows the full regression output.

### Entailment trials

**Figure 4.**
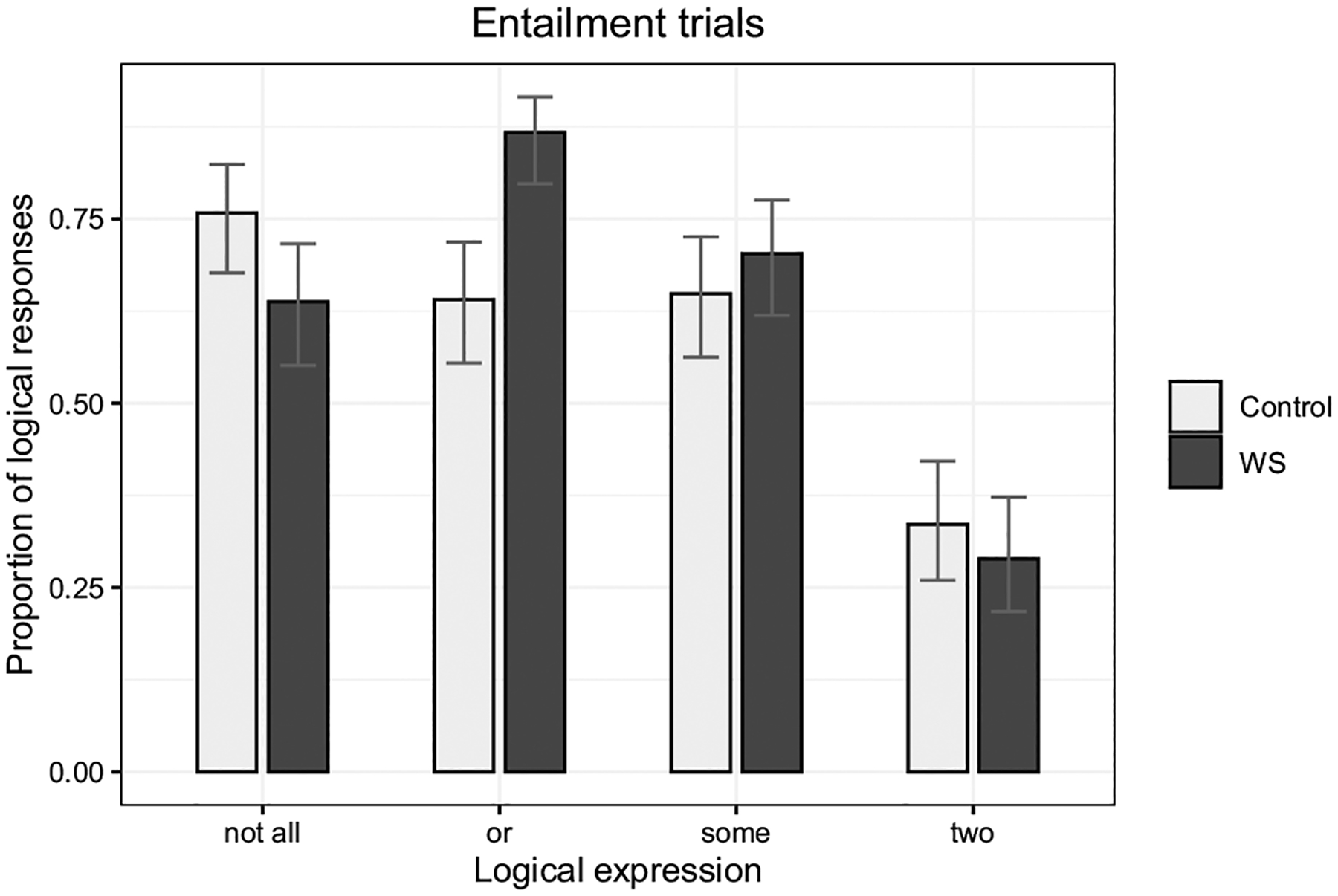
Proportion of semantic responses for the WS and control groups by logical expression. Error bars represent 95% binomial confidence intervals.

Unlike truth-conditional trials, where the answer could either be correct or incorrect, in entailment trials, all the answers were technically correct, and our question was which of the two interpretations (logical or pragmatically enriched) participants chose. In an event where three out of three clouds turned gray, and the statement “Some of the clouds will turn gray” was uttered, participants could give either a “yes” response to the question “Am I right?” (accessing a logical interpretation of the quantifier *some* “some and possibly all”) or a “no” response (accessing a pragmatically enriched interpretation “some but not all”). Our dependent measure was whether participants interpreted the scalar term logically. Except for the term *all*, for each scalar pair (<*not all, none*>, <*or, and*>, <*some, all*>, <*two, three*>), participants completed four trials, where a weaker term on the scale was used in a situation where the stronger term also applies. Results for all terms across the WS and control groups are shown in Figure 4. Using a binomial logistic regression model, we calculated whether group and the type of logical expression predicted the type of interpretation (logical or pragmatically enriched). Both independent variables were treatment-coded: the control group and the quantifier *some* were set as reference levels, respectively. A maximally converging random effects structure included random intercepts for participants and items. The model with an interaction term for group vs. logical expression failed to converge, we therefore report a model with main effects only. The analysis revealed no effect of group (*b* = 0.16, *SE* = 0.29, *z* = 0.54, *p* = .587). Participants were more likely to accept a logical interpretation for the disjunction operator *or* as “one of the objects and possibly both” (*b* = 0.45, *SE* = 0.21, *z* = 2.13, *p* = .033) and less likely to accept a logical interpretation of *two* as “two and possibly more than two” (*b* = −1.83, *SE* = 0.21, *z* = −8.57, *p* = .587; full model output, as well as model syntax can be found in the Appendix, Table B.4).

Since the model with the interaction term did not converge, we proceed to the analysis of group differences for each logical term individually. We first report the results for the <*some, all* > scale, considered to be a staple of analysis in the linguistic literature. Here, we ask whether participants accepted the *some* statement in a context where *all* is true. We maintain that accessing this reading reveals knowledge of entailment relations. In a binomial mixed-effects logistic regression, we regressed the type of response (logical vs. pragmatically enriched) against participant group (WS vs. control); the model included random intercepts for participants.^10^ We used treatment coding for the factor group, setting the reference level to the control group. The analysis revealed no difference between the WS and the control group for *some* on the log-odds of choosing a logical interpretation (control group: 65%, WS: 70%; β = 0.137, *SE* = 1.862, *z* = 0.074, *p* = .941). We observed a similar result for the other logical expressions. There was no significant effect of group for *not all* (Control 76%, WS: 64%; β = −1.002, *SE* = 1.687, *z* = −0.606, *p* = .545), the logical operator *or* (Control 64%, WS: 87%; β = 1.530, *SE* = 1.964, *z* = 0.779, *p* = .436), or the numeral *two* (Control 34%, WS: 29%; β = −0.433, *SE* = 1.820, *z* = −0.238, *p* = .812). Thus, we registered no difference between WS speakers and the control group in their choice of interpretation for any of the logical expressions.

**Figure 5.**
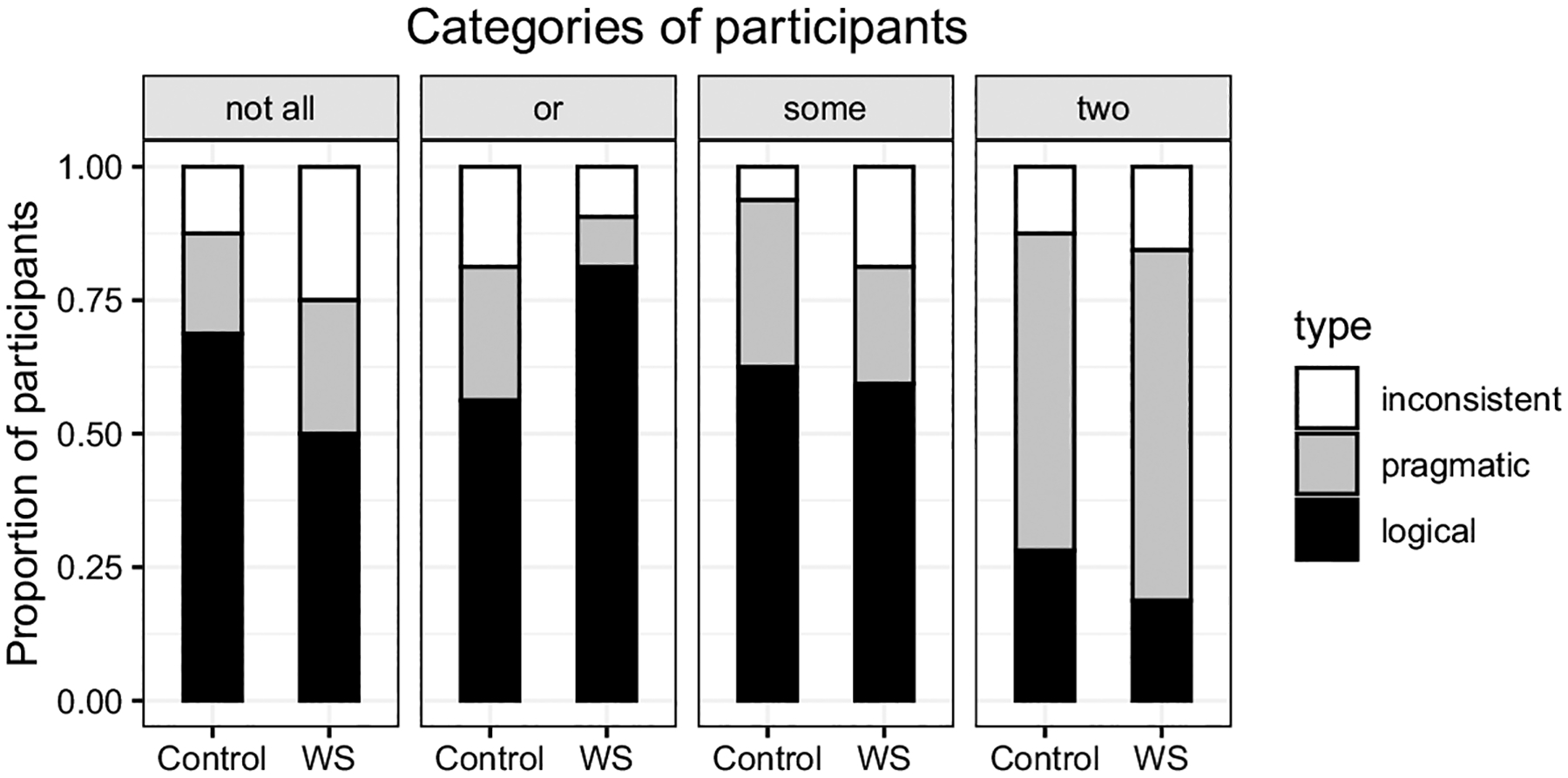
Proportion of participants who interpreted logical expressions logically vs. pragmatically. Participants interpret the term logically, i.e., they respond “yes” to the question “Am I right” to statements with a weaker term, such as *some*, in situations where a statement with a stronger term, such as *all*, is true. Participants interpret the term pragmatically when they reject the statement (respond “no”) to the use of a weaker term in the same situation.

While Figure 4 shows the average proportion of logical interpretations for different scales and groups, it does not reveal whether participants are consistent in their interpretations, i.e., whether they always choose a logical or a pragmatically enriched interpretation in entailment trials for each of the terms. The average proportions also do not show whether the consistency patterns align for the WS and control groups. In order to explore the consistency of responses, we categorized all participants as favoring a logical or a pragmatically enriched (pragmatic) interpretation: if a participant always chose the logical interpretation (four trials out of four possible), we categorized them as “logical,” if they chose zero logical readings, we categorized them as “pragmatic.” Participants who gave from one to three logical readings were assigned to the “inconsistent” category (Figure 5).

We performed Fisher exact tests of independence to assess the association between group (WS vs. control) and participant type (logical, pragmatic, or inconsistent) for each of the logical expressions. We found no significant association between group and participant type for the quantifiers *some* (*p* = .3) and *not all* (*p* = .27), the logical operator *or* (*p* = .115), or for the numeral *two* (*p* = .762).

### Effect of demographic variables on the interpretation of logical expressions in entailment trials

To evaluate whether age or KBIT scores contributed to the logical vs. pragmatic preferences of people with WS, we fitted the data with a binomial logistic regression with the type of response (logical vs pragmatically enriched) as the dependent variable and age in years and raw KBIT verbal and non-verbal scores as continuous predictors. The model also included random intercepts for participants and items. For the in-lab cohort, age was not a significant predictor of whether a participant preferred semantic or pragmatic responses in the entailment trials (*b* = −0.06, *SE* = 0.10, *z* = −0.66, *p* = .510). Neither were the verbal (*b* = −0.04, *SE* = 0.05, *z* = −0.80, *p* = .424) nor non-verbal KBIT scores (*b* = −0.06, *SE* = 0.07, *z* = 0.79, *p* = .430; see the model syntax and the regression output in the Appendix, Table B.5). For the online cohort, we again found no effect of age (*b* = 0.03, *SE* = 0.03, *z* = 0.89, *p* = .373), verbal (*b* = 0.0, *SE* = 0.02, *z* = −0.25, *p* = .801), or non-verbal scores (*b* = −0.05, *SE* = 0.04, *z* = −1.32, *p* = .186; Table B.6).

### Discussion

In Experiment 1, we assessed whether speakers with WS show core knowledge of logical expressions, as well as entailment patterns associated with them. The analysis of truth-conditional trials revealed that speakers with WS performed well above chance in this task, reaching more than 80% of correct responses even for quantifiers that involve negation (*not all*). The quantifier *not all* is likely difficult for speakers with WS since it contains negation, and negation has been shown to be more challenging for individuals with WS, particularly when it interacts with other logical operators (Musolino et al., 2010). More generally, converging evidence suggests that processing negation requires additional cognitive resources compared to the processing of affirmative sentences in neurotypical adults (Clark & Chase, 1972; Horn, 1989; Just & Carpenter, 1971; Kaup & Dudschig, 2020) and children (Nordmeyer & Frank, 2014). Furthermore, cross-linguistic evidence suggests that complex quantifiers, such as *not all*, are rarely (if ever) lexicalized suggesting that their meaning is harder to acquire than the meaning of simple quantifiers, such as *some* or *all* (Horn, 1972; Katzir & Singh, 2013).

While participants performed generally well above chance, people with WS were less accurate than the control group of adults on truth-conditional trials (WS group: 93%, control group: 99%). In the WS group, we found a link between their KBIT verbal scores and the probability of choosing the correct answer. It is not entirely clear whether there is a causal link between KBIT scores, which generally reflect vocabulary and conceptual development, and the performance on our tasks. It is possible that performance on the tasks that involve logical operators and tasks that tap into open class vocabulary items can be affected by a third variable, such as working memory capacity. However, our experimental design does not allow us to establish this causal link.

The analysis of entailment trials revealed that speakers with WS not only know the basic truth conditions associated with a variety of abstract logical expressions, but they also show patterns of preferences for logical vs. pragmatically enriched readings that are attested in the neurotypical adults who served as a control group. Access to the logical reading of a scalar term implies that these speakers must know that when a sentence like “*All* clouds will turn gray” is true, “*Some* clouds will turn gray” must also be true, and that when a sentence like “A star *and* a puzzle will appear on the screen” is true, “A star *or* a puzzle will appear on the screen” must also be true. This, in turn, shows that they have a deep understanding of the set-theoretic properties of English quantifiers and other types of logical expressions, since entailment relations follow from these properties. What renders this conclusion even more interesting is the fact that, on an everyday basis, people rarely use quantifiers like *some*, for example, in situations where *all* would be more appropriate. In other words, due to the pragmatic bias, it would be rather odd indeed for someone to say “Look, some of the circles are red,” whereas in fact, all the circles are red. This intuition has been assessed and verified experimentally (van Tiel et al., 2021).

In Experiment 1, some individuals with WS consistently interpreted logical expressions logically (as did some of the neurotypical adults). However, their acceptance of the logical interpretation may simply stem from the fact that they do not recognize that these logical expressions have stronger alternatives. Lacking access to relevant alternatives might be one of the reasons of increased acceptance of logical interpretations, as evidence from acquisition experiments with TD children suggests (Barner et al., 2011; Gotzner et al., 2018, 2020). This hypothesis predicts that when given a choice of a weaker and a stronger term in a situation that licenses the use of the stronger term, some individuals with WS might be at chance when asked to identify which of the terms is felicitous. In Experiment 2, we evaluate this hypothesis, asking whether speakers with WS correctly identify the strength of alternatives for logical expressions. Specifically, we ask whether, given a choice of two possible descriptions of a scene where a stronger term holds, speakers with WS in fact prefer the stronger term (as should be shown by neurotypical adults).

## Experiment 2: relative strength of logical expressions

### Participants

The same group of participants (32 WS speakers and 32 neurotypical adult controls) took part in the current experiment. They always performed Experiment 2 after Experiment 1. Data from one neurotypical adult were excluded due to a technical error, thus data from 31 participants in the control group were included in the analysis.

### Design, materials, and procedure

We tested participants’ knowledge of the felicity conditions (Austin, 1962) associated with the use of logical expressions tested in Experiment 1 using a felicity judgment task (Chierchia et al., 2001) where participants usually hear two descriptions of the same scene, and their task is to pick the better description. The use of a term is judged felicitous if it satisfies the pragmatic principles, such as the maxim of quantity that prescribes speakers to be as informative as they can be (Grice, 1975).

Mirroring the design of Experiment 1, participants were tested on four pairs of logical expressions: <*some, all*>, <*or, and*>, <*not all, none* > and <*two, three* >. For the scales <*some, all*>, <*not all, none*>, and <*two, three*>, participants were presented with arrays of objects displayed on a computer monitor. A woman’s face then appeared on the left side of the screen and described the situation using a prerecorded statement containing one of the two terms on a given scale. Then, a man’s face appeared on the opposite side of the screen, and a prerecorded statement was played, containing the other term on the scale. Both faces remained on the screen along with text bubbles containing the terms that each had used. Finally, a third prerecorded voice asked “Which is it, *___ or ___* ?” A sample dialogue for the scale <*some, all* > is shown in (9).

(9) Sample conversation for Experiment 2, scalar pair <*some, all* >.
(a) Man’s voice: I say, some of the trees are green.
(b) Woman’s voice: I say, all of the trees are green.
(c) Third voice: Which is it, *some* or *all*?

To make the trials more natural, we modified the prompts and order of presentation for the logical expressions <*or, and*>, as we did for Experiment 1. Example (10) shows a sample conversation for the <*or, and* > pair.

(10) Sample conversation for Experiment 2, scalar pair *and*>.
(a) Man’s voice: I say, a cake and a cross will appear on the screen.
(b) Woman’s voice: I say, a cake or a cross will appear on the screen.
(c) Third voice: Which is it, *or* or *and*?

**Figure 6.**
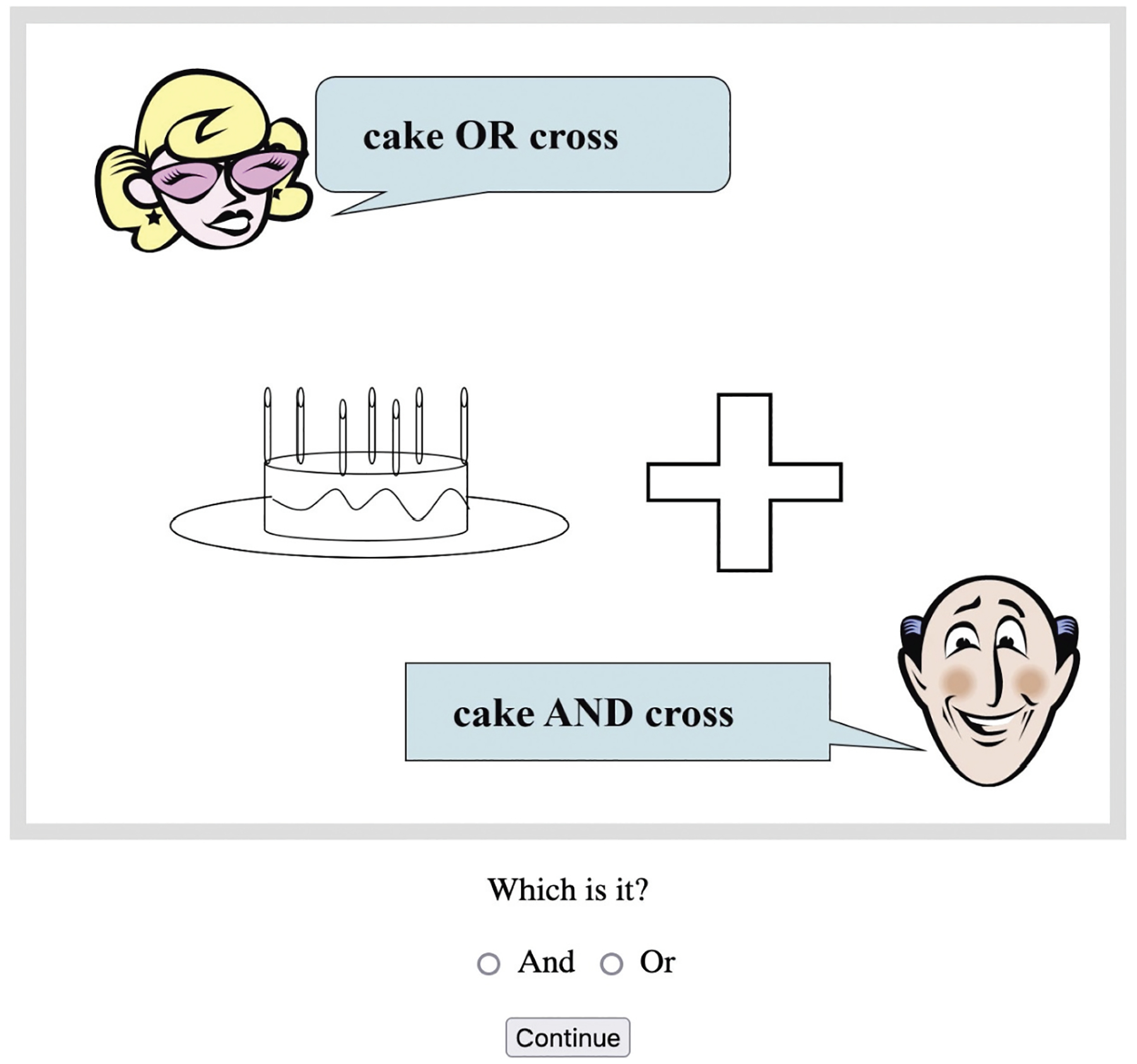
Experiment 2 trial. The woman’s voice says: “I say, a cake or a cross will appear on the screen.” The man’s voice says: “I say, a cake and a cross will appear on the screen.” Third voice poses the question: “which is it, *or* or *and*?” Participant’s task is to select the better term.

In this conversation, the male and female faces appeared before any of the two relevant objects did and the prerecorded statements consisted in predictions^11^ regarding what would appear on the screen. A sample trial is shown below for the pair <*or, and* > in Figure 6.^12^

**Figure 7.**
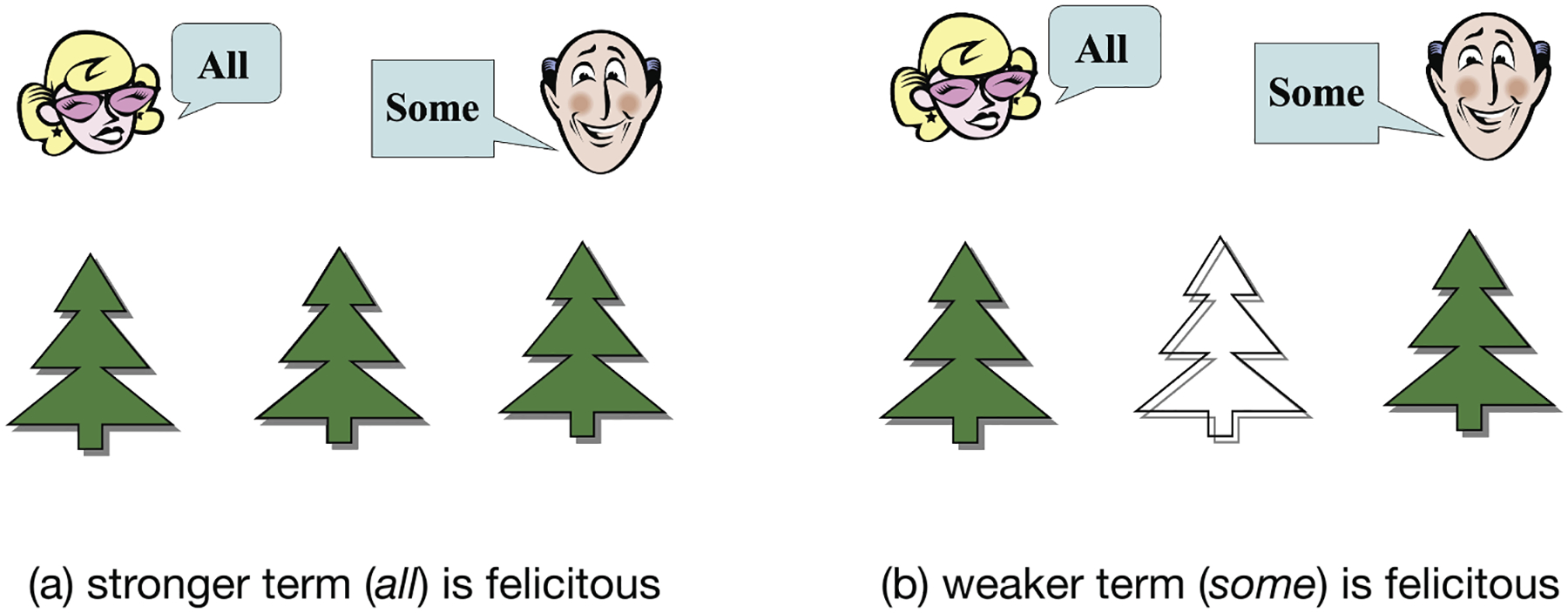
Sample trials for Experiment 2. The woman’s voice says: “All of the trees are green.” The man’s voice says: “Some of the trees are green.” The third voice asks: “Which is it, *some* or *all*?” Panel “a” shows a trial where the stronger term on the scale (in this case, *all*) is felicitous. Panel “b” shows a trial where the weaker term (in this case, *some*) is felicitous.

In Experiment 2, we presented a weaker term on the scale with a stronger alternative – a set-up that allowed us to evaluate the knowledge of felicity conditions. For each scalar pair, participants were asked to evaluate eight different statements, four of which were designed to make the use of the stronger term felicitous, e.g., *all* as in Figure 7, panel “a,” and four of which were designed to make use of the weaker term felicitous, e.g., *some* as in Figure 7, panel “b.”

The trials where the stronger term on the scale was felicitous (Figure 7, panel “a”) evaluated the knowledge of scale strength. When an utterance with a stronger term, such as *all*, is true, a parallel statement with a weaker term, such as *some* is also true, however *all* is more felicitous. These trials show whether a participant recognizes a stronger term in contexts where both apply. The second type of trial (Figure 7, panel “b”) tests whether participants recognize that this entailment relation is not symmetric. If a statement with a weaker term is true, it does not entail that a stronger statement is also true. For example, the truthfulness of *some* statements does not entail the truthfulness of *all* statements. To complete the experiment, participants had to evaluate a total of 32 statements (4 terms × 8 statements). Trials were blocked by the pair of logical expressions (e.g., <*some, all*>), the order of blocks and trials within the blocks was randomized, as well as the order of query within each pair. The position of the woman’s face (left) and the man’s face (right) on the screen was fixed, who spoke first, and which speaker used the more felicitous of the two terms were fully counterbalanced.

### Results

**Figure 8.**
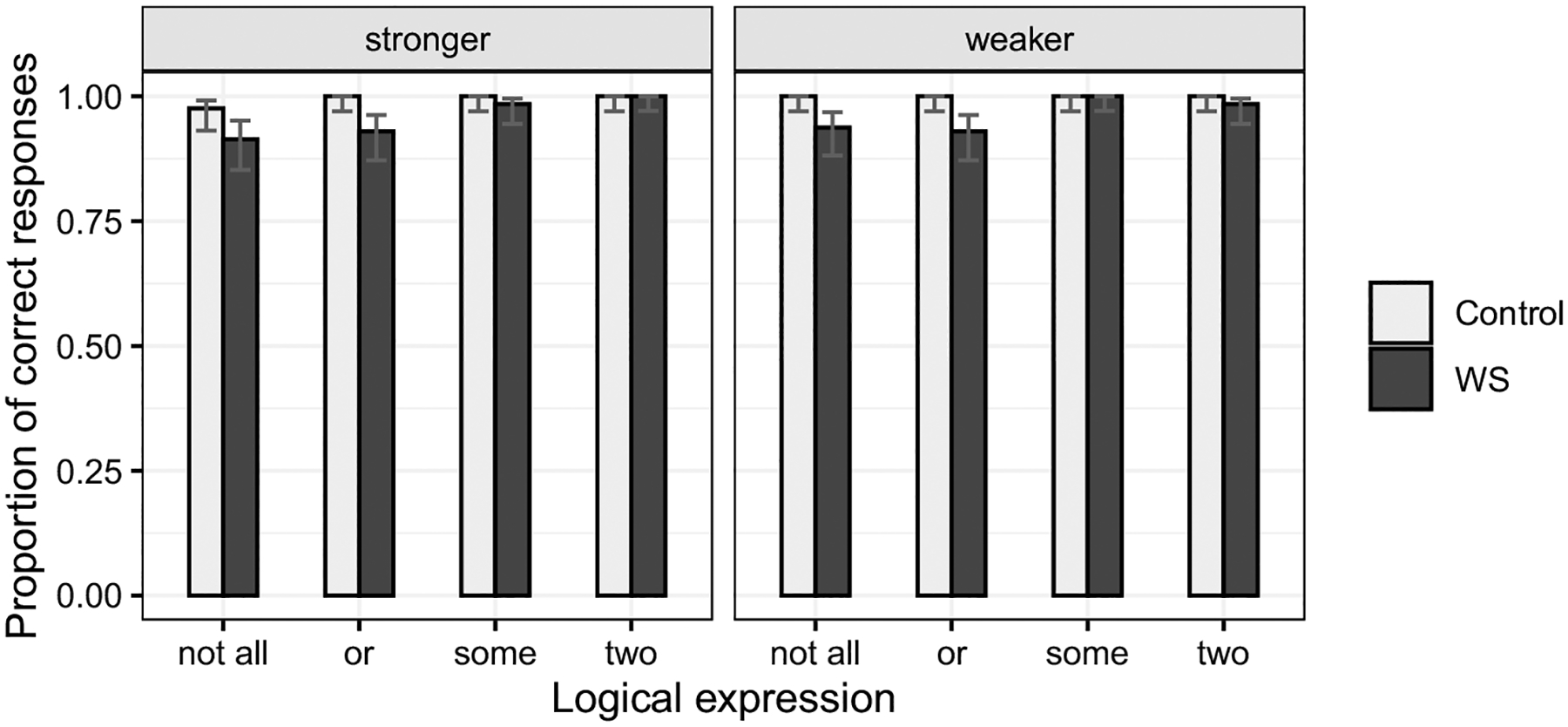
Proportion of correct responses for the WS and control groups by logical expression. Error bars represent 95% binomial confidence intervals.

We observed ceiling performance both for the WS group and the control group: speakers with WS chose the stronger term correctly more than 91% of the time, with performance on the numeral pair <*two, three* > reaching 100% correct (Figure 8, left panel). Performance for the trials where the weaker term was felicitous was also at ceiling, with more than 92% of correct responses for each of the logical expressions (Figure 8, right panel). Since a model with the independent fixed effects for group, type of context (weaker vs. stronger), and type of logical expression did not converge, we first ran a simpler binomial mixed-effects model with type of context (weak or strong) and group, as well as their interaction, as independent variables, and the type of response (correct vs. incorrect) as a binomial dependent variable. Since the interaction term did not improve the model fit (likelihood ratio test, χ^2^(1) = 3.58, *p* = .058), we report results of a model with main effects only. Contrasts for both independent variables were treatment coded with reference levels set to the control group and the stronger contexts, respectively. A binomial mixed-effects regression with random intercepts for participants and items showed that individuals with WS were statistically less accurate than TD adults (WS: 96% of correct responses; TD adults 99.7%; β = −2.76, SE = 1.15, z = −2.40, *p* = .017). Performance in this experiment was not different for contexts that favored weaker vs. stronger terms (b = 0.30, SE = 0.54, z = 0.57, *p* = .570; full model output can be found in the Appendix, Table B.7).

We further aimed to evaluate whether group membership (WS vs. control) and type of logical expression (<*not all, none*>, <*or, and*>, <*some, all*>, <*two, three*>) affected the probability of a correct response. We fitted the data with a binomial mixed-effects model with group and logical expression as independent variables, and response as the binomial dependent variable. Including an interaction term was not possible due to convergence issues possibly caused by at-ceiling performance in the control group and the associated extremely low variance. As in the previous model, we applied treatment coding to the factor group setting the control group as the reference level. We used sum coding for the factor logical expression to compare the performance on each of the logical expression to an overall probability of correct responses. The analysis confirmed that participants with WS scored fewer correct responses than the control group (b = −2.80, SE = 1.15, z = −2.42, *p* = .015); however, their overall rate of correct responses was very high (96%). In fact, only five individuals had the overall correct rate between 78% and 84%, all other individuals scored above 90%. There were small but significant differences between logical expressions: the rate of correct responses was the highest for the pairs <*some, all* > and <*two, three*>; (99.6%), and slightly lower for <*not all, none* > and <*or, and*> (95.6% and 96.4%, respectively). Since all of these results were at ceiling, we do not interpret these differences further. The full regression model output can be found in the Appendix, Table B.8.

To further examine whether there was between-participant variation in the WS group, we pooled weaker and stronger trials together to get a value that reflects their understanding of the asymmetry of the entailment patterns. We calculated for each participant and each scalar term whether they performed above chance (7 correct responses out of 8 trials for each term, null hypothesis can be rejected at *p* < .05 with a binomial exact test). In the control group, all participants performed above chance for the scales <*some, all*>, <*and, or*>, and <*two, three* >. For <*not all, none* > 97% of neurotypical adults (30 out of 31 participants) performed above chance. For *not all* and *or*, 84% of WS speakers (27 of 32 participants) performed significantly above chance. Among those, 72% (23 of 32 participants) responded to all trials with *not all* correctly and 78% (25 of 32 participants) to all trials with *or* correctly. For the numeral *two* and the scalar *some* these numbers reached 97% and 100% (31 and 32 of 32 WS participants). Overall, these results suggest that most speakers with WS correctly recognize the strength of logical expressions for a variety of scales.

### Effect of demographic variables on the probability of correct responses

We ran a binomial mixed-effects logistic regression treating the type of response (correct vs. incorrect) as the dependent variable and the verbal and non-verbal KBIT scores as independent variables. The maximally converging random effect structure included random intercepts for participants and items. As for Experiment 1, we report the results of regression modeling separately for the different cohorts; however, the patterns we observed were similar. For the in-lab cohort, the model with verbal and non-verbal scores as predictors resulted in a singular fit, possibly reflecting low variance in the data, making estimates unreliable (see the model output in the Appendix, Table B.9). For the online cohort, verbal scores were a significant predictor of response (*b* = 0.08, *SE* = 0.03, *z* = 3.00, *p* < .01), while there was no significant effect of non-verbal scores (*b* = 0.01, *SE* = 0.07, *z* = 0.17, *p* = .865; Table B.10). The model with AGE as an additional continuous predictor did not converge.

### Discussion

We evaluated (a) whether speakers correctly identify a stronger term on a scale when given two members of that scale in contexts where the stronger term applies and (b) whether they recognize that statements where only a weaker term applies do not entail statements with a stronger term. Most speakers with WS showed near-ceiling scores on the task, indicating their developed pragmatic competence in the domain of scalar expressions: they correctly identified the stronger term on the scale in contexts that permitted the use of both terms and rejected the stronger term in contexts that only allowed the use of a weaker term. People with WS were less likely to choose the correct scalar terms than adults, even though in absolute numbers the difference between average scores was small. As in Experiment 1, verbal but not non-verbal scores significantly affected the log-odds of choosing a correct response.

## General discussion

In this paper, we asked whether people with WS show knowledge of the semantic computations necessary for the understanding of natural language quantifiers, numerals, and logical operators. We predicted that people with WS should perform significantly above chance if they possess this type of linguistic knowledge. Our investigation was motivated by the modularity debate and was designed to probe the nature of semantic computation in people with WS. We asked whether it is possible for learners to acquire subtle and deep linguistic principles in the face of mild to moderate intellectual deficits as well as severe cognitive deficits in spatial and numerical cognition. If so, this would then lend support to the modularity perspective on the human mind, in particular, by showing that deep understanding of the semantics of logical terms is present despite severe deficits in other aspects of cognition (Fodor, 1983). Alternatively, if the linguistic system of individuals with WS lacks these deep principles, we would expect them to interpret sentences with quantifiers, numerals, and logical operators differently than our control groups. One possibility is that they would not be able to comprehend these complex structures and that their performance in our tasks would not be different from chance.

In our experiments, individuals with WS showed a firm understanding of the truth conditions associated with each of the logical expressions we tested, as evidenced by their well-above-chance performance. The terms that involve negation appeared more challenging for a subset of the participants, presumably due to increased cognitive load associated with processing negation (cf. Musolino et al., 2010). Moreover, when quantifiers and other logical expressions could be interpreted either logically or pragmatically, participants with WS displayed similar interpretive preferences to those of neurotypical adults. We further showed that the acceptance of logical interpretations could not be explained by a lack of understanding of the strength of logical expressions. Individuals with WS consistently correctly identified the contexts where stronger terms are felicitous, showing understanding of their relative strength. In both Experiments 1 and 2, we observed performance significantly above chance for scales that involve quantifiers, numerals, and logical connectors. We take these results as evidence for representations of the abstract meanings associated with logical expressions, challenging some arguments in the literature that people with WS have “superficial” semantic representations. Our results suggest that deep understanding of meaning is possible for speakers with WS at least in some domains: components of quantifier logic, numerosity, and disjunction, and their expression in language.

Is it possible that high performance in our tasks does not reflect abstract principles of semantic computation, but rather some other mechanisms? Neuroconstructivist writers have offered an alternative explanation for high levels of performance in a variety of linguistic tasks among people with WS. One proposal is that strong performance on language tasks is caused by and reflects strong auditory memory (Karmiloff & Karmiloff-Smith, 2001) which masks the underlying deficits in representations, such as containing less abstract information and more perceptually based detail (Thomas & Karmiloff-Smith, 2003, p. 652). We would then hypothesize that such auditory memory strength could also allow people with WS to appear to have mastered the understanding of logical expressions without the deployment of abstract principles. While the advocates of neuroconstructivism do not articulate exactly *how* strong auditory memory could allow a speaker to acquire the meaning of, for example, quantifiers, we might imagine that speakers would map the individual words, such as *some* and *all* to situations in which they are used. The speakers would further notice that words, such as *some*, are used in two different types of situations where they are taken to mean “some but not all” and “some and possibly all.” After all, ambiguity is a common feature of the lexicon, and such learning should be at least in principle possible. However, we maintain that good auditory memory is unlikely to enable people with WS to acquire the semantic meaning of words like *some* that in some contexts mean “some and possibly all,” since at least for some quantifiers it is attested that speakers rarely use these terms with their logical meaning, i.e., they do not use *some* in situations when *all* is true (van Tiel et al., 2021). To be sure, experiments that use stimuli extracted from a corpus of Twitter posts reveal that quantificational terms are interpreted as *pragmatically enriched* rather than logically (semantically) most of the time (Sun et al., 2024). Additional evidence for the relative infrequency of logical meaning compared to the pragmatically enriched one comes from ERP-studies that report a larger P300 effect for semantic readings of logical expressions, indicating that a pragmatically enriched reading is normally expected (Holtgraves & Kraus, 2018). If other logical expressions are also primarily used with their pragmatically enriched (rather than logical) meanings, we would have expected people with WS to interpret these expressions pragmatically. But as our results have shown, they can access both the logical and the pragmatically enriched interpretations.

In comparison to the neurotypical adults who reached 99% correct in Experiment 1, individuals with WS performed less successfully (86% correct for the negative quantifier *not all*; 90% or better for the other logical terms). A similar performance asymmetry in tasks involving disjunction and negation (Musolino & Landau, 2010) has been reported, and some (Thomas et al., 2010) have interpreted these data as a sign of atypical development, consistent with the neuroconstructivist approach. However, Musolino and Landau (2010, 2012) emphasize that understanding linguistic development in people with WS does not reduce to assigning “typical” vs. “atypical” labels. Rather, they see the goal of scientific explanation as spelling out the mechanisms of language development in a population with an atypical cognitive profile. The modularity view here predicts that individuals with deficits in some cognitive domains may nevertheless show the same computational principles in language as appears in neurotypical individuals. This view relies on clear and detailed linguistic theories of how these computational principles give rise to sentence meaning involving logical elements. Without knowledge of such principles, we would expect to see chance performance in tasks that involve the understanding of quantifiers, numerals, and logical operators.

We note that our results, which indicate clear successes among WS participants in interpreting logical terms, might appear to conflict with reported deficits in other lexical-semantic abilities in this group (e.g., Romero-Rivas et al., 2023). For example, adolescents with WS have been reported to struggle with differentiating lies from ironic jokes, and some have suggested this reflects deficits in pragmatic reasoning (Sullivan et al., 2003). Parent self-reports confirm this observation: parents of Spanish speakers with WS have shown deficits in irony and joke comprehension, as well as ambiguity resolution more generally (Sepúlveda & Resa, 2024). Perhaps more pertinent to our current studies, individuals with WS perform significantly worse on tasks that involve relational words, compared to words that refer to concrete entities (Mervis & John, 2010). Acquisition of logical expressions, under this view, should be particularly challenging due to their abstract semantics, which are not easily inferred from superficial usage patterns.

Our findings raise the question of how an individual with WS would be able to acquire the complex and abstract meanings of logical expressions. We propose that acquisition of the meanings of these expressions by people with WS takes place in the same way as for typically developing individuals. Specifically, both groups have the capacity to use relations between sets as the basis for constructing representations of a range of logical terms. Rather than memorizing specific uses of these terms, speakers can rely on their capacity to reason about set relations and rely on their understanding of set relations to identify the semantic meanings of logical expressions. Thus, for example, knowing that a property applies to all individuals allows speakers to infer that this property applies to a subset of those individuals as well. And understanding set relations then allows people to derive entailment patterns. Speakers can then rely on their knowledge of entailment patterns to interpret quantifiers in a variety of contexts including those that they may have never encountered in the input.

However, a solid understanding of set relations alone might be insufficient to acquire the meanings of logical expressions in a given language. The set of possible meanings vastly surpasses the number of meanings that are actually lexicalized in any given language. These lexicalization patterns have been explained by universal semantic and pragmatic constraints (Horn, 1972; Katzir & Singh, 2013), as well as learnability considerations (Steinert-Threlkeld & Szymanik, 2019, 2020; van de Pol et al., 2019; Van De Pol et al., 2023). If learners with WS had different learnability constraints due to their altered neural development, they might assign different patterns of meaning to logical expressions. Our results speak against this hypothesis. Rather, it appears that universal semantic, pragmatic, and learnability constraints that operate in neurotypical learners also allow people with WS to acquire the same types of meanings. For example, these constraints make lexicalizing meanings, such as “some but not all” for *some* or “one of the objects but not both” for *or*, not plausible. Learners would then expect that words, such as *some* would be compatible with both a logical and a pragmatically enriched reading. These universal constraints would then explain how learners acquire the logical meaning of these expressions in the absence of sufficient input.

We further maintain that the contrast between strong performance in our current studies and the weak performance in other studies of semantics (reviewed in the Background section and above) stems from differences in the kinds of linguistic knowledge that have been assessed in the two cases. Our investigation has targeted the principles of semantic computation within a framework that formalizes the underlying representations needed for understanding logical expressions, while most previous work arguing for semantically shallow representations has focused on the associative connectedness of the lexicon and the structure of concepts. Quantifiers, numerals, and logical operators that have been the focus of our paper, belong to the class of logical expressions, and their complexity lies in the type of meaning that they capture: they do not refer to objects but rather denote properties of sets and relations between sets. Interpreting sentences with these terms thus involves complex semantic computation, as has been independently demonstrated in theoretical linguistics. Our results suggest that semantic computation is in fact spared in individuals with WS as evidenced by their understanding of sentences that involve quantificational and other abstract logical terms. In other words, people with WS develop the same competencies as TD children do. We see the development of these competencies as evidence that people with WS follow a typical language acquisition path where they develop the same mechanisms of meaning computation, albeit proceeding at a slower pace, as it has been argued for morphosyntax (Musolino & Landau, 2012) and indeed, for other cognitive domains such as spatial and numerical development (Landau & Hoffman, 2012). For example, many fundamental spatial functions are preserved in individuals with WS (Landau et al., 2006), even if their developmental trajectories are more extended than in typically developing children, and full mastery is achieved at a later chronological age (Landau & Ferrara, 2013). Notably, a speculative hypothesis about the WS developmental trajectory for both language and space is that development proceeds slowly and – at least for some domains – is subject to “arrest” during adolescence (Landau & Hoffman, 2012). This would result in robust acquisition of milestones that are typically acquired early in development, but only fragile (or absent) acquisition of milestones typically acquired much later in development.

The developmental literature on typical language acquisition lends support for this hypothesis, as the understanding of set relations, the concept of entailment, and the understanding of negation are all in place by 3 years of age in neurotypically developing children. That is, they constitute milestones that are typically acquired “early.” For example, Feiman et al. (2019) demonstrated that children’s understanding of novel number terms is constrained by understanding of numerical entailment relations in 2- and 3-year-olds, revealing that they have grasped the concept of entailment. The study of numerical relations further reveals that children as young as two can reason about sets – a core capacity for grasping the meaning of quantifiers. Children further learn to count small sets by the age of 3.5 (Gelman et al., 1978; Wynn, 1990). Developmental studies also show that children show early evidence of understanding negation as a logical concept, with the first negative forms appearing between 15 months (Frank et al., 2017) and 19 months (Bloom, 1970). Children understand logical negation from the age of 2 (Austin et al., 2014; Feiman et al., 2017), with some studies registering understanding of logical negation already at 18 months of age (de Carvalho et al., 2021).

The literature on the comprehension of quantifiers in typically developing children further shows that children as young as 2 years of age show a core understanding of quantifier meaning, with this ability developing gradually throughout preschool years (Barner et al., 2009; Katsos et al., 2016). For example, 5-year-olds and, according to some studies, 8–10-year-olds (Noveck, 2001) have not fully mastered the adult-like ability to reject the use of weaker scalar terms in contexts that license stronger terms (Katsos et al., 2011; Papafragou & Musolino, 2003).^13^ Studies on the acquisition of numerals and quantifiers further suggest that even 3-year-old children differentiate between these two related classes of expressions and have an adult-like preference for the exact interpretation of number terms (e.g., *three* as “exactly three”) at this age but somewhat take longer to acquire a preference for a pragmatically enriched interpretation of quantifiers (interpreting *some* as “some but not all”). Interestingly, our WS participants also show sensitivity to this difference between numerals and quantifiers: they rarely accepted the logical interpretation of numerals (interpreting *two* as “at least two”) but were eager to accept *some* when it meant “some and possibly all,” similarly to what we observed in neurotypical adults (who accepted the semantic interpretation for *some* 65% of the time).^14^ In other words, our participants with WS showed fine-grained understanding of the meaning of quantifiers, numerals, and logical operators, including the fact that they differ in their tendencies to be interpreted logically or pragmatically. Each of these aspects of meaning is also acquired by typically developing children fairly early in development, generally before the age of 7 (Guasti et al., 2005). As we argued in the Background section, the fact that such knowledge is already present in young children does not suggest that the principles underlying semantic computation are simple.

The “developmental delay and arrest” account predicts that along with knowledge of quantifiers and other logical terms, individuals with WS should also show deep linguistic knowledge in other core linguistic domains that mature early. Indeed, research on language in individuals with WS suggests that many basic properties of language are preserved in this population, even if such properties are acquired at a later chronological age than in typically developing (Landau & Hoffman, 2012). For example, in the domain of determiner acquisition (words, such as *a* and *the* in English), speakers with WS either display adult patterns of use for the determiner or a delay in that pattern’s appearance, but no difference in the way they use the definite determiner (Modyanova, 2009). This result is notable because determiner meaning is highly abstract, and competence in this domain is a sign of highly abstract semantic representations. These findings are consistent with studies showing mastery of core syntactic and morphological properties of language in speakers with WS (Clahsen & Almazan, 1998; Musolino et al., 2010). The developmental delay and arrest hypothesis further predicts that individuals with WS may never reach full competence for linguistic phenomena that mature in adolescence in typically developing children. The study on differentiation of ironic jokes from lies brings supporting evidence: Sullivan et al. (2003) notes that speakers with WS often failed to differentiate ironic jokes from lies. At the same time, their pattern of errors was like the one of younger children rather than speakers with acquired brain damage.

This developmental account clearly points to a much-needed clarification in the literature on WS as well as the literature on other neurodivergent populations: this concerns what one might mean by “atypical.” Of course, this term is usually contrasted with “typical,” which refers to the averages (and variance) at different chronological ages, computed from (usually standardized) developmental measures age. On this criterion, yes, people with WS are “atypical” in the sense that they appear to achieve linguistic goals at a later chronological age than is reported in the broader neurotypical population. But there is another criterion which we believe is crucial in explaining how development emerges across different kinds of experience, and indeed, different cognitive disorders. This criterion concerns the *nature of knowledge that is achieved* for any cognitive domain. The study of people’s knowledge of logical terms is one example, but there are many other domains in which development may be slow – even very slow – but this developmental process reaches an end-state which represents highly abstract understanding and, to our knowledge, is not qualitatively different from that of the broader “typical” population. It is this latter sense of “typical” that we are after in our studies of people with WS and the linguistic principles that guide neurotypical adults’ use of logical forms. We believe that we have shown that subtle and nuanced aspects of linguistic quantifiers are present in people with WS and that that knowledge is for all intents and purposes no different from that found in neurotypical adults.

In sum, we find converging evidence from cognitive development and language acquisition studies that core functions that are acquired early in typical development also successfully develop in people with WS even though at a later chronological age. The picture we have painted here stands at odds with the neuroconstructivist account which predicts that the atypically developing brain should have broad effects on cognition (Karmiloff-Smith, 1998). Under this view, we would have expected to see severe deficits in the comprehension of words with abstract and complex meaning, with quantifiers, numerals, and logical operators being likely areas where such deficits would manifest themselves. We have provided empirical evidence that this prediction does not hold. At the same time, the evidence we have presented also challenges the traditional argument for modularity that rested on the asymmetry between spatial and numerical skills on the one side and linguistic skills on the other side. It appears now that all of these skills develop more slowly in WS but do so following a slower version of the typical developmental trajectory. Our findings show that the cognitive capacities necessary for the acquisition of quantifiers, numerals, and logical operators are clearly present in people with WS at some developmental point. It is an open question whether they go through the same developmental stages as neurotypical children do in acquiring these terms. However, given the fact that non-linguistic number understanding in WS also shows signature core abilities that develop to a similar level to that shown by much younger typically developing children (Libertus et al., 2014), complete separation of function (non-linguistic, linguistic) may be generally unlikely in the case of language acquisition in WS.

However, separation of function is only one of the criteria for modular systems. Fodor (1983) emphasized that not all of the criteria need to apply at the same time for a system to be considered modular. In our introduction, we suggested that robustness under deficit is one of the criteria that is most relevant to the study of linguistic skills in WS. We have now demonstrated that deep linguistic knowledge emerges in WS individuals despite their level of intellectual impairment. This knowledge also emerges relatively early in typically developing children, consistent with the idea that the brain/mind has dedicated neural and cognitive structures for the acquisition of complex linguistic patterns and that these structures can emerge even if there exist deficits in other cognitive domains – a hallmark of modularity.

Advocates of neuroconstructivism have repeatedly emphasized that atypically developing individuals may reach a neurotypical adult level of performance on some tasks, albeit by taking alternative paths and recruiting other, possibly non-linguistic, resources. However, there is at present no competing account that could explain how the comprehension of sentences with logical expressions, such as quantifiers, could develop in a different way without the recruitment of concepts such as logical entailment and the understanding of set relations. Furthermore, having demonstrated the complexity of underlying computations spelled out within the theoretical linguistic literature, we maintain that early development of a linguistic ability does not imply simplicity of underlying representations and computation.

## Conclusion

The question of whether people with WS show “intact” linguistic abilities lies at the heart of the modularity/neuroconstructivism debate. Karmiloff-Smith et al. (1997) have argued that if “intactness” is viewed as a property of language development, under no circumstance could we argue that language development in WS is intact – simply because their levels of acquisition are like those of much younger typically developing children. This is true if we believe that age-of-acquisition is a uniquely critical fact for determining whether there is any a priori foundation for mastering a given property of linguistic structure. In this paper, we have taken another approach to language and cognition in WS and evaluated “intactness” in terms of the computational principles required for complex tasks and their intersection with different developmental timelines between individuals with WS and typically developing individuals (Landau & Hoffman, 2012; Musolino & Landau, 2010; Musolino et al., 2010). We have demonstrated that adolescents and adults with WS show evidence of deep and abstract semantic principles that underlie the semantic computations necessary for the understanding of quantifiers, numerals, and logical operators. Thus, WS presents a case study showing the acquisition of sophisticated linguistic knowledge despite substantial cognitive deficits in other domains. Our data further revealed that the overall level of cognitive abilities and verbal skills in particular contributed to performance on some of our tasks; this is not surprising but is hardly diagnostic of a “difference” in what has been acquired. We look forward to future research which carefully delineates the various factors which underlie the acquisition of quantifier structure and use, both in children and adults who meet the age criterion for “typical,” and who do not.

## Supplementary Material

Supp 1

Supplemental data for this article can be accessed online at https://doi.org/10.1080/15475441.2026.2702526.

## Figures and Tables

**Table 1.** Structure of the experiment. For each logical expression, we created three possible outcomes. For *all*, all of the outcomes create truth-conditional trials. For other expressions, two of the outcomes constitute truth-conditional trials, and one outcome constitutes an entailment trial.

	*all*			*not all*			*some*			*or*			*two*		
outcome	0	2	3	0	2	3	0	2	3	0	1	2	0	2	3
correct response	no	no	yes	y/n	yes	no	no	yes	y/n	no	yes	y/n	no	yes	y/n
	tr	tr	tr	ent	tr	tr	tr	tr	ent	tr	tr	ent	tr	tr	ent

**Table 2.** Entailment trials. Possible responses and the corresponding interpretation of each of the logical expressions.

	not all	or	some	two
outcome	0	2	3	3
**response: “yes”**	not all and possibly none	one of the objects or possibly both	some and possibly all	two and possibly more
reading: logical				
**response “no”**	not all but not none	one of the objects but not both	some but not all	two but not more than two
reading: pragmatically enriched				

## Data Availability

Data and analysis code are available at an anonymous OSF repository: https://osf.io/s7t6b/overview?view_only=a1c7cb41e1e940eea96e75c6394069d5 Demographic data has been removed from the data set to protect the privacy of our participants.

## Footnotes

1 Mental age is a metric developed to capture the relationship between chronological age and cognitive abilities in typically developing individuals of different ages. Scores are determined by performance on standardized cognitive abilities tests, which then yield normed scores that can be compared with the scores of atypically developing children. The use of mental age as a measure of cognitive abilities has been long criticized in psychometric and human rights literature (Rogers & McGuire, 2024) largely because they are relatively superficial.

2 See Dolscheid and Penke (2018) for a study of quantification in WS and Down syndrome that uses a “give-me-quantifier” task.

3 An alternative “restricted quantification view” defines the meaning of quantifiers as a type of determiner that specifies how to apply a predicate to a restricted domain denoted by the first set, rather than a term that denotes relations between two sets (Knowlton et al., 2023). On this view, *some* indicates that the statement in (1b) expresses an existential proposition and that instructs speakers how to apply the predicate “received an A” to a restricted domain “students.”

4 The term “felicitous” was originally introduced by Austin (1962) to capture conditions on the use of utterances that go beyond truth conditions, the latter referring to the state of the world under which a given sentence is true (Montague, 1973; Tarski, 1944). A use of a word or a sentence is termed felicitous if it follows pragmatic principles, such as the maxim of quantity prescribing speakers to be informative (Grice, 1975).

5 For the in-lab study we recruited US undergraduates and we did not collect information about their specific ages.

6 Predictions were used here because scalar implicatures are thought to be computed less often in such contexts, compared to regular descriptive contexts, such as in (1b) (Musolino & Lidz, 2006; Noveck, 2001; Papafragou & Musolino, 2003). This phenomenon might be related to less strict requirements on knowledgeability of the speaker for prediction contexts: if the speaker does not have complete evidence about the actual state of the world they describe, they might not have the information whether a stronger term on the scale applies, and therefore the implicature is not computed (Goodman & Stuhlmüller, 2013; Katsos, 2008). Using the prediction context thus allowed us to create a linguistic environment where the logical meaning is more accessible.

7 For the term *all*, all three outcomes, where either none, two, or all three objects undergo the change, are truth-conditional since *all* represents the strongest term on the scale <*some, all* >.

8 The term *all* does not appear in this table since all of the trials for this term are truth-conditional: statements with *all* are not ambiguous, since *all* is the stronger term on the scale.

9 We chose to use raw Verbal and Non-verbal scores because our question is whether absolute level of language and/or non-linguistic ability predict performance. We examined age in the regression as well.

10 A model with a random slope resulted in non-convergence, we therefore dropped random slopes from the random effect structure. We further had to exclude random intercepts for items due to close to zero variance.

11 Predictions were used for <*or, and*> scale to make trials more natural. As discussed for Experiment 1, prediction contexts may bias the interpretation of a weaker scalar term toward a logical meaning. However, even in that case a stronger term on the scale is more felicitous. Participants both in the control and WS groups overwhelmingly chose correct answers in this condition. In other words, they correctly picked the “better” description, as we instructed them.

12 We recognize that this set up requires participants to understand that the relevant logical terms represent alternative utterances that could be used to describe the situation. The near-ceiling performance suggests that participants indeed had the correct understanding of the tasks. Had they not associated logical terms with alternative utterances, we would have expected them to pick the terms randomly. The data clearly show that it was not the case.

13 Guasti et al. (2005) suggest that under appropriate experimental conditions children at the age of 7 display adult-like preferences in the interpretation of scalar terms.

14 We specifically used prediction contexts to make the semantic interpretation more easily accessible. Our results should not be interpreted as a failure to compute scalar implicature, since scalar implicature is standardly computed less often in such contexts.
