# Supplementary material for: Logic and felicity in the face of intellectual disability: linguistic scales and Williams syndrome
Source: Lang Learn Dev. Author manuscript; Available in PMC 2026 Aug 1. (PMC13426231; doi:10.1080/15475441.2026.2702526)
Supplement: Supp 1 [file NIHMS2197353-supplement-Supp_1.docx]

**Appendix A**

***Effect of cohort***

**Experiment 1.** In this section, we ask whether data from different cohorts could be analyzed jointly, or whether performance across cohorts differed asking for a cohort-specific analysis. For truth-conditional data, we fitted a binomial logistic regression model with participants and items as random effects, cohort and group as independent variables, and the type of response (correct or incorrect) as the dependent variable. Contrast for group and cohort here and in other models were treatment-coded with reference levels set to the control group and the lab cohort, respectively. The analysis revealed no interaction between participant group (WS vs. control) and cohort (β = −0*.*362*, SE* = 1*.*128*, z* =−0*.*320*, p* = 0*.*749). The interaction term did not improve the model fit (χ^2^(1) = 0*.*106*, p* = 0*.*749). A simpler model with main effects for groups and WS cohorts showed no effect of cohort (β = −0*.*332*, SE* = 0*.*635*, z* = −0*.*523*, p* = 0*.*601).

The analysis of entailment trials confirmed this result. We treated group and cohort as independent variables and interpretation (logical vs. pragmatic) as a binomial dependent variable. Random effect structure included random intercepts for participants. We were not able to include random effects for items due to convergence issues. The interaction between group and cohort was not significant (β = 0*.*823*, SE* = 0*.*563*, z* = 1*.*461*, p* = 0*.*144). The inclusion of the interaction term in the model did not improve its fit compared to a model that only contained main effects (χ^2^(1) = 2*.*105*, p* = 0*.*147). A model with main effects for group and cohort revealed no significant effect of cohort (β = −0*.*472*, SE* = 0*.*285*, z* = −1*.*654*, p* = 0*.*098). In sum, there was neither a clear effect of cohort, nor an interaction between participant group and cohort in our data. This result serves as a study-internal replication of our findings showing the robustness of the patterns that we registered.

**Experiment 2.** For Experiment 2, we also used a binomial logistic regression with random intercepts for participants and items to evaluate the effect of cohort on whether participants selected the correct term (binomial dependent variable). Neither the interaction between participant group and cohort (β = 23.77*, SE* = 24*.*84*, z* = 0*.*957*, p* = 0*.*339), nor the cohort itself assessed in a simpler model without the interaction (β = −1*.*437*, SE* = 1*.*128*, z* = −1*.*274*, p* = 0*.*203) revealed an effect of cohort. Table A.1 shows age metrics and KBIT scores for WS participants for both cohorts.

| Cohort | Age (years; months) | | | KBIT verbal | | | KBIT nonverbal | | |
| --- | --- | --- | --- | --- | --- | --- | --- | --- | --- |
|  | *mean* | *median* | *SD* | *mean* | *median* | *SD* | *mean* | *median* | *SD* |
| in-lab | 16; 4 | 16; 1 | 3; 0 | 41.4 | 44 | 6.24 | 21.7 | 21.5 | 4.03 |
| online | 23; 10 | 19; 5 | 10; 11 | 57.0 | 59.5 | 18.8 | 23.0 | 22 | 6.84 |
| Overall | 21; 0 | 17; 7 | 9; 5 | averages not meaningful^[[1]](#footnote-1)^ | | | 22.5 | 21.5 | 5.9 |

Table A.1: Age and KBIT information (WS). The in-lab cohort completed KBIT (Kaufman & Kaufman 1990). The online cohort completed KBIT-2 (Kaufman & Kaufman, 2004). Reported scores are raw scores.

**Appendix B**

***Experiment 1. Truth-conditional trials***

Model syntax:

glmer (score ~ logical expression + group + (1|participant) + (1|item))

| Term | estimate | *SE* | *z* | *p* | 95% CI |
| --- | --- | --- | --- | --- | --- |
| (Intercept) | 4.99 | 0.30 | 16.39 | < .001*** | [4.39, 5.58] |
| all | 0.11 | 0.24 | 0.46 | .644 | [-0.37, 0.59] |
| not all | -1.09 | 0.23 | -4.65 | < .001*** | [-1.55, -0.63] |
| or | 0.36 | 0.30 | 1.21 | .228 | [-0.22, 0.94] |
| some | -0.14 | 0.27 | -0.54 | .593 | [-0.67, 0.38] |
| two | 0.76 | 0.33 | 2.31 | .021* | [0.12, 1.41] |
| group | -2.20 | 0.30 | -7.47 | < .001*** | [-2.78, -1.63] |

Table B.1 Regression output for a binomial mixed-effects model for truth-conditional trials. We applied sum coding for the factor logical expression, and treatment coding for the factor group, the reference level is the control group. We selected a maximal random effect structure that still converged. The model with logical term x group interaction did not converge, possibly due to zero variance in some cells.

Model syntax:

glmer (score ~ age + KBIT verbal + KBIT non-verbal + (1| participant) + (1 |item))

| Term | estimate | *SE* | *t* | *p* | 95% CI |
| --- | --- | --- | --- | --- | --- |
| (Intercept) | -0.64 | 1.02 | -0.63 | .526 | [-2.64, 1.35] |
| age | -0.11 | 0.04 | -3.05 | .002** | [-0.18, -0.04] |
| kbit_verbal_raw | 0.10 | 0.02 | 4.42 | < .001*** | [0.06, 0.15] |
| kbit_nonverbal_raw | 0.08 | 0.05 | 1.69 | .091 | [-0.01, 0.17] |

Table B.2 WS group, in-lab cohort, KBIT first edition. Regression output for a binomial mixed-effects model for truth-conditional trials.

Model syntax:

glmer (score ~ age + KBIT verbal + KBIT non-verbal + (1| participant) + (1 |item))

| Term | estimate | *SE* | *z* | *p* | 95% CI |
| --- | --- | --- | --- | --- | --- |
| (Intercept) | -0.36 | 0.84 | -0.43 | .670 | [-2.01, 1.29] |
| age | -0.10 | 0.03 | -3.06 | .002** | [-0.16, -0.03] |
| kbit_verbal_raw | 0.11 | 0.02 | 5.29 | < .001*** | [0.07, 0.15] |
| kbit_nonverbal_raw | 0.01 | 0.04 | 0.31 | .760 | [-0.06, 0.08] |

Table B.3 WS group, online cohort, KBIT-2 (second edition). Regression output for a binomial mixed-effects model for truth-conditional trials.

***Experiment 1. Entailment trials***

Model syntax:

glmer (interpretation type ~ logical expression + group + (1|participant) + (1|item))

| Term | estimate | *SE* | *z* | *p* | 95% CI |
| --- | --- | --- | --- | --- | --- |
| (Intercept) | 0.80 | 0.24 | 3.37 | .001*** | [0.34, 1.27] |
| WS | 0.16 | 0.29 | 0.54 | .587 | [-0.41, 0.72] |
| not all | 0.12 | 0.21 | 0.59 | .552 | [-0.28, 0.53] |
| or | 0.45 | 0.21 | 2.13 | .033* | [0.04, 0.87] |
| two | -1.83 | 0.21 | -8.57 | < .001*** | [-2.24, -1.41] |

Table B.4 Regression output for a binomial mixed-effects model for entailment trials. Factor group is treatment-coded with the reference level set to the control group; factor logical expression is treatment-coded as well with the reference level set to *some*. A more complex model with an interaction term for group x logical expression, as well as a model with a random slope for logical expression per participant, failed to converge.

Model syntax:

glmer (interpretation type ~ age + kbit_verbal_raw + kbit_nonverbal_raw+ (1|id) + (1|item))

| Term | estimate | *SE* | *z* | *p* | 95% CI |
| --- | --- | --- | --- | --- | --- |
| (Intercept) | 2.05 | 2.83 | 0.72 | .470 | [-3.50, 7.59] |
| age | -0.06 | 0.10 | -0.66 | .510 | [-0.25, 0.13] |
| kbit_verbal_raw | -0.04 | 0.05 | -0.80 | .424 | [-0.13, 0.05] |
| kbit_nonverbal_raw | 0.06 | 0.07 | 0.79 | .430 | [-0.08, 0.20] |

Table B.5 WS group, in-lab cohort, entailment trials, KBIT (first edition). Regression output for a binomial mixed-effects model for entailment trials.

Model syntax:

glmer (interpretation type ~ age + kbit_verbal_raw + kbit_nonverbal_raw+ (1|id) + (1|item))

| Term | estimate | *SE* | *z* | *p* | 95% CI |
| --- | --- | --- | --- | --- | --- |
| (Intercept) | 1.48 | 0.98 | 1.52 | .128 | [-0.43, 3.40] |
| age | 0.03 | 0.03 | 0.89 | .373 | [-0.03, 0.08] |
| kbit_verbal_raw | -0.00 | 0.02 | -0.25 | .801 | [-0.04, 0.03] |
| kbit_nonverbal_raw | -0.05 | 0.04 | -1.32 | .186 | [-0.13, 0.03] |

Table B.6 WS group, online cohort, entailment trials, KBIT-2 (second edition). Regression output for a binomial mixed-effects model for entailment trials.

***Experiment 2.***

Model syntax:

glmer (response ~ group + context type + (1|id) + (1|item))

| Term | estimate | *SE* | *z* | *p* | 95% CI |
| --- | --- | --- | --- | --- | --- |
| (Intercept) | 8.63 | 1.45 | 5.95 | < .001*** | [5.78, 11.47] |
| groupWS | -2.76 | 1.15 | -2.40 | .017* | [-5.02, -0.50] |
| context type | 0.30 | 0.54 | 0.57 | .570 | [-0.75, 1.36] |

Table B.7 Regression output for a binomial mixed-effects model for Experiment 2. Factor group is treatment-coded with the reference level set to the control group; factor context type is treatment-coded with the reference level set to the contexts where the stronger term is more felicitous. A more complex model with an interaction term for group x context type did not improve the model fit compared to the model with main effects only.

Model syntax:

glmer (response ~ group + logical expression + (1|id) + (1|item))

| Term | estimate | *SE* | *z* | *p* | 95% CI |
| --- | --- | --- | --- | --- | --- |
| (Intercept) | 8.99 | 1.44 | 6.25 | < .001*** | [6.17, 11.81] |
| WS | -2.80 | 1.15 | -2.42 | .015* | [-5.06, -0.53] |
| not all/none | -1.45 | 0.33 | -4.43 | < .001*** | [-2.09, -0.81] |
| or/and | -1.17 | 0.33 | -3.53 | < .001*** | [-1.83, -0.52] |
| some/all | 1.31 | 0.58 | 2.27 | .023* | [0.18, 2.44] |
| two/three | 1.31 | 0.58 | 2.27 | .023* | [0.18, 2.44] |

Table B.8 Regression output for a binomial mixed-effects model for Experiment 2. Factor group is treatment-coded with the reference level set to the control group; factor logical expression is sum-coded. A more complex model with an interaction term for group x logical expression, as well as a model with a random slope for logical expression per participant failed to converge.

Model syntax:

glmer (score ~ kbit_verbal_raw + kbit_nonverbal_raw + (1|id))

| Term | estimate | *SE* | *t* | *p* | 95% CI |
| --- | --- | --- | --- | --- | --- |
| (Intercept) | -79.35 | 37.19 | -2.13 | .033* | [-152.23, -6.46] |
| kbit_verbal_raw | 2.31 | 1.00 | 2.32 | .020* | [0.36, 4.26] |
| kbit_nonverbal_raw | 0.22 | 0.28 | 0.78 | .436 | [-0.33, 0.78] |

Table B.9 WS group, in-lab cohort, KBIT (first edition). Regression output for a binomial mixed-effects model for experiment 2.

Model syntax:

glmer (score ~ kbit_verbal_raw + kbit_nonverbal_raw + (1|id))

| Term | estimate | *SE* | *z* | *p* | 95% CI |
| --- | --- | --- | --- | --- | --- |
| (Intercept) | 0.04 | 1.59 | 0.02 | .982 | [-3.07, 3.14] |
| kbit_verbal_raw | 0.08 | 0.03 | 3.00 | .003** | [0.03, 0.13] |
| kbit_nonverbal_raw | 0.01 | 0.07 | 0.17 | .865 | [-0.13, 0.15] |

Table B.10 WS group, online cohort, KBIT-2 (second edition). Regression output for a binomial mixed-effects model for experiment 2.

1. The ranges of scores for KBIT and KBIT-2 are different, therefore averaging over these scores will produce misleading results. [↑](#footnote-ref-1)
